# Mutation-Structure-Function Relationship Based Integrated Strategy Reveals the Potential Impact of Deleterious Missense Mutations in Autophagy Related Proteins on Hepatocellular Carcinoma (HCC): A Comprehensive Informatics Approach

**DOI:** 10.3390/ijms18010139

**Published:** 2017-01-11

**Authors:** Faryal Mehwish Awan, Ayesha Obaid, Aqsa Ikram, Hussnain Ahmed Janjua

**Affiliations:** Department of Industrial Biotechnology, Atta-ur-Rahman School of Applied Biosciences (ASAB), National University of Sciences and Technology (NUST), H-12 Islamabad 44000, Pakistan; faryal_mehwish@yahoo.com (F.M.A.); ayesha_obaid_nust@live.com (A.O.); aqsa_ikram@yahoo.com (A.I.)

**Keywords:** autophagy related proteins, missense single nucleotide polymorphisms, post-translational modifications, hepatocellular carcinoma, computational analysis

## Abstract

Autophagy, an evolutionary conserved multifaceted lysosome-mediated bulk degradation system, plays a vital role in liver pathologies including hepatocellular carcinoma (HCC). Post-translational modifications (PTMs) and genetic variations in autophagy components have emerged as significant determinants of autophagy related proteins. Identification of a comprehensive spectrum of genetic variations and PTMs of autophagy related proteins and their impact at molecular level will greatly expand our understanding of autophagy based regulation. In this study, we attempted to identify high risk missense mutations that are highly damaging to the structure as well as function of autophagy related proteins including LC3A, LC3B, BECN1 and SCD1. Number of putative structural and functional residues, including several sites that undergo PTMs were also identified. In total, 16 high-risk SNPs in LC3A, 18 in LC3B, 40 in BECN1 and 43 in SCD1 were prioritized. Out of these, 2 in LC3A (K49A, K51A), 1 in LC3B (S92C), 6 in BECN1 (S113R, R292C, R292H, Y338C, S346Y, Y352H) and 6 in SCD1 (Y41C, Y55D, R131W, R135Q, R135W, Y151C) coincide with potential PTM sites. Our integrated analysis found LC3B Y113C, BECN1 I403T, SCD1 R126S and SCD1 Y218C as highly deleterious HCC-associated mutations. This study is the first extensive in silico mutational analysis of the LC3A, LC3B, BECN1 and SCD1 proteins. We hope that the observed results will be a valuable resource for in-depth mechanistic insight into future investigations of pathological missense SNPs using an integrated computational platform.

## 1. Introduction

Autophagy is a tightly regulated ubiquitous catabolic process for the degradation of cytoplasmic proteins and organelles via lysosome, particularly during starvation or stress [1]. Proteins encoded by autophagy-related genes (ATGs) are the core machinery in the control of autophagy [2]. Several biological approaches have revealed physiological roles and pathological effects of autophagy, including quality control of intracellular proteins, suppression of tumour formation, innate immunity, antigen presentation etc. [3]. Unique features and functions of liver and due to its crucial role in normal metabolic functions to which autophagy is intimately connected has made it an important organ for autophagy research [4]. Deletion models of ATGs in liver have provided evidence of its significance in homeostasis, stress, energy conservation, cellular differentiation and development. For example, alteration of lysosomal degradation in hepatocytes has been observed after inhibition of autophagy in mouse models, resulting in a fourfold increase in liver weight [5]. Similarly, nutrient starvation experiments in mice revealed autophagy induced degradation of 40% of total proteins in the liver during a 48 h time period [6]. These data reveals the significant contribution of autophagy in the cellular maintenance of hepatic functions.

Autophagy not only plays an important role in normal liver physiology, but has also been previously associated with fatty and alcoholic liver diseases, viral hepatitis, cirrhosis, fibrosis and hepatocellular carcinoma (HCC) [7,8]. The functional role of autophagy in HCC has been explored and studied extensively over the past few years. Based on the recent findings, autophagy is considered to play a dual role in HCC, both as an anti-cancerous and pro-cancerous [7]. As manipulation of autophagy can significantly affect the HCC treatment efficacy, therefore, understanding the underlying molecular mechanisms is critical to develop novel therapeutics targeting autophagy to cure liver cancer. Four autophagy-related markers were included in the current study, including microtubule-associated proteins 1A/1B light chain 3A (LC3A), microtubule-associated proteins 1A/1B light chain 3B (LC3B), beclin-1 (BECN1) and stearoyl-CoA desaturase-1 (SCD1), as they have been previously reported to be potential markers for estimating HCC tumour progression and metastasis [7,9,10,11,12].

Under stress conditions, autophagy is dynamically induced for cell survival and homeostasis, supported by fast and reversible post-translational modifications (PTMs) in autophagy related proteins [13]. PTMs in autophagy related proteins have been emerging as highly significant regulators with phosphorylation being the most intensively studied PTM, followed by ubiquitination and acetylation in the autophagy process [14]. Dysregulation in the PTM sites has been previously reported to cause serious pathological conditions . In addition to PTMs, mutations in genes encoding part of the autophagy machinery are known to abolish autophagy and cause various pathological conditions [15]. Single nucleotide polymorphisms (SNPs) are responsible for the majority of genetic variation in the human genome [16]. To date approximately 5,000,000 SNPs have been discovered in the coding region of human proteins responsible for genetic variation [17]. Although many mutations are phenotypically neutral, missense mutations often have serious damaging effects on protein structure and function. As missense mutations are found in coding regions of the proteins, therefore they can alter the structure, stability and function of proteins ultimately leading to various serious human diseases. 

Recently, large-scale studies have shown that overlap between PTMs and SNPs resulted in damaged PTMs which have profound impact on both gene and protein function, and they are associated with human cancer. A damaged PTM can be defined as a PTM site that bears a SNP. In various studies, damaged PTMs have been studied in greater detail and it has been shown that mutation of serine (Ser) to arginine (Arg) at residue position 215 occurring on a potential PTM site in TP53 is associated with breast cancer [18]; another example is the mutation of T286 in cyclin D1 causing the loss of phosphorylation resulting in its nuclear accumulation in esophageal cancer [19]. In another study, a genome-wide analysis of SNPs revealed that around 70% of reported non-synonymous SNPs (nsSNPs) might potentially influence the protein phosphorylation status and play a key role in rewiring the biological pathways [20]. Moreover, Radivojac et al. found correlation between phosphorylation site disrupting variants and somatic cancer mutations. The authors observed significant enrichment of disease-associated mutations in phosphorylation sites. Their findings revealed that mutations causing gain and loss of phosphorylation sites are linked to human cancer [21]. Several PTM-related SNPs have been identified in recent findings. 4195 phosphoproteins were collected by Yang et al. with 15,738 phosphorylation sites and 1515 SNPs in the flanking phosphorylation sites. The authors revealed that mutations in 64 phosphorylation sites resulted in disease phenotypes [22]. Ryu et al. utilized Swiss Variant database and collected 33,651 variants. The authors predicted the effects of variants on phosphorylation sites [23]. Ren et al. identified 64,035 phosphorylation-related SNPs in 17,614 proteins by mapping SNPs onto sequences from RefSeq Build. The authors used NCBI dbSNP and collected 91,797 nsSNPs [20].

**Figure 1:**
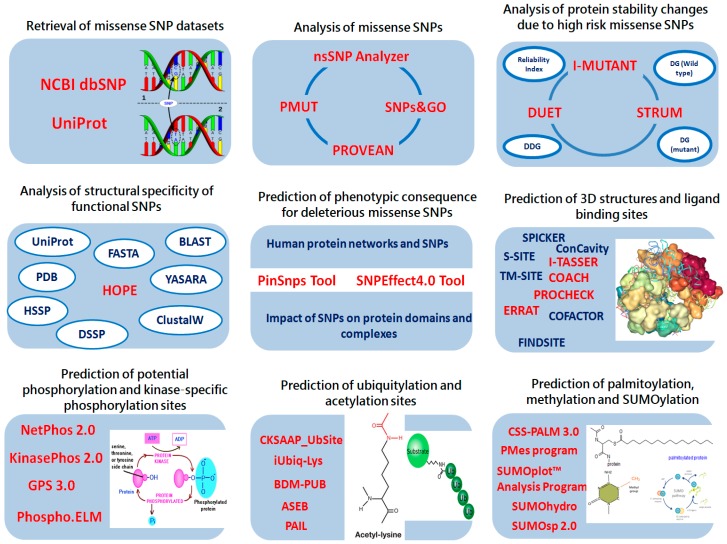
Schematic representation of prioritization pipeline. Identification of the most deleterious mutations in LC3A, LC3B, BECN1 and SCD1 based on various in silico tools. Abbreviations used: DG, free energy change; DDG, the predicted free energy change value.

In addition to mutations affecting PTM sites, SNPs alone particularly in ATGs have mounting evidences of direct involvement in human diseases, including cancers [24,25]. The study conducted by Kang et al. indicated that mutations in ATGs may contribute to cancer development by deregulating the autophagy process [26]. Similarly, the study conducted by An et al. revealed that somatic mutation and loss of expression of ATG5 gene might play a role in the pathogenesis of gastric cancer and HCC by altering autophagic and apoptotic cell death [27]. Another study conducted by Qin et al. on Chinese population revealed that two SNPs in ATG10 were significantly associated with altered risk of breast cancer [28]. The recent advancements and developments in high throughput sequencing technologies have increased the rate at which genetic variations are identified. Furthermore, the comprehensive availability of such sequencing data is enabling researchers to extensively use bioinformatics tools in order to extract useful hidden clinical information. There is a need for highly accurate and efficient method to uncover pathogenic and deleterious SNPs from the readily accessible pool of variant data, and to further explore their impact at the molecular level. In silico tools can be effectively utilized for prioritizing SNPs in cost efficient manner and to further investigate structural consequences of disease-causing mutations. A comprehensive integrative bioinformatics analysis of the functional and structural impact of highly damaging SNPs will be highly supportive and will also facilitate in finding true disease associations. In the present study, our goal was to understand and predict highly damaging missense SNPs and potential PTM sites as well as potential crosstalk among PTM sites and SNPs to explore the mutation-structure-function relationship in autophagy related proteins LC3A, LC3B, BECN1 and SCD1 (see Figure 1). We proposed that the LC3B Y113C, BECN1 I403T, SCD1 R126S and SCD1 Y218C mutations induced major phenotypic damages in LC3B, BECN1 and SCD1 proteins, altering their structural behavior, which might play an important role in inducing HCC.

## 2. Results

### 2.1. Missense SNP Datasets

Polymorphism data for the *LC3A*, *LC3B*, *BECN1* and *SCD1* genes were obtained from the NCBI dbSNP database and the UniProt database. In our data search, we cross-checked the variant information available in UniProt and NCBI dbSNP; removed invalid variants based on the erroneous sequences and alignments, and removed the overlapping data. As a result, a total of 28 missense SNPs in *LC3A*, 52 in *LC3B*, 145 in *BECN1* and 117 in *SCD1* gene were considered for further analysis. To determine whether a given missense mutation affected the functions of respective genes, we subjected the missense mutations to a variety of in silico SNP prediction algorithms.

### 2.2. Missense SNP Analysis

Four in silico SNP prediction algorithms were employed in our analysis including nsSNP Analyzer, PROVEAN, PMUT, and SNPs & GO. According to nsSNP Analyzer results, in LC3A, 16 missense SNPs cause disease, whereas 12 missense SNPs are neutral (Table 1). In LC3B, 15 missense SNPs cause disease and 37 missense SNPs are neutral (Table 1). In BECN1, 45 SNPs cause disease, whereas 100 SNPs are neutral and in SCD1, 55 SNPs cause disease and 62 SNPs are neutral (Table 1). PMUT predicted that 15 SNPs are pathological and 13 SNPs are neutral in LC3A, in LC3B, 27 SNPs are pathological and 25 SNPs are neutral, in BECN1, 77 SNPs are pathological and 68 SNPs are neutral whereas in SCD1, 45 SNPs are pathological and 72 SNPs are neutral (Table 1). According to PROVEAN, in LC3A, 21 SNPs were considered deleterious and 7 as neutral (Table 1). In LC3B, 39 were predicted to be deleterious and 13 being neutral (Table 1). In BECN1, 72 were predicted to be deleterious and 73 being neutral (Table 1). In SCD1, 42 SNPs were considered deleterious and 75 as neutral (Table 1). Findings of SNPs & GO algorithm predicted that 17 SNPs cause disease and 11 SNPs are neutral in LC3A (Table 1). In LC3B, 22 SNPs were predicted to cause disease and 30 being predicted to be neutral (Table 1). In BECN1, 40 SNPs were predicted to cause disease and 105 being predicted to be neutral (Table 1). In SCD1, 77 SNPs were predicted to cause disease and 40 being predicted to be neutral (Table 1). 8 SNPs in LC3A (R24C, P55L, R70C, F79V, F79S, K49A, K51A and G120A), 12 SNPs in LC3B (R11C, P32L, R37Q, G40C, R68A, R70A, R70H, F79S, D106G, Y113S, Y113C and G120A), 20 SNPs in BECN1 (L112R, S113R, R164C, L194P, 255C, R292C, R292H, E302K, L314H, Y338C, C353Y, C375R, I403S, I403T, W425C, F431V, F123A, D133A, Y352A, W425A) and 18 SNPs in SCD1 (Y88C, G89R, T100I, R121C, H125P, R126S, R131W, R135W, M144T, Y151C, Y218C, W238G, W238R, G272R, R283W, F323V, C326G, G331S) were found to be deleterious by all four SNP prediction algorithms. As different criteria and parameters were used by each algorithm to evaluate the SNPs, SNPs with more positive results are more likely to be truly deleterious. Here, we classified SNPs as high-risk if they were observed to be deleterious by three or more than three SNP prediction algorithms. 16 SNPs in LC3A, 18 in LC3B, 40 in BECN1 and 43 in SCD1 (Table 1) met these criteria and were chosen for further analysis. The selected state-of-the-art tools have covered maximum number of methods (alignment scores, neural networks, hidden Markov models, support vector machine and Bayesian classification) used for the prediction of highly deleterious SNPs.

### 2.3. Conservation Profile of High-Risk Missense SNPs

Amino acids play an essential role in various biological activities specifically those located in enzymatic sites or involved in protein–protein interactions (PPI) and are likely to be more conserved than other residues. Furthermore, SNPs that are present at highly conserved positions tend to be more damaging than SNPs being positioned at non-conserved sites. To further examine the potential effects of the high-risk SNPs in LC3A, LC3B, BECN1 and SCD1, we analyzed the conservation pattern at all amino acid sites with high risk SNPs.

**Figure 2:**
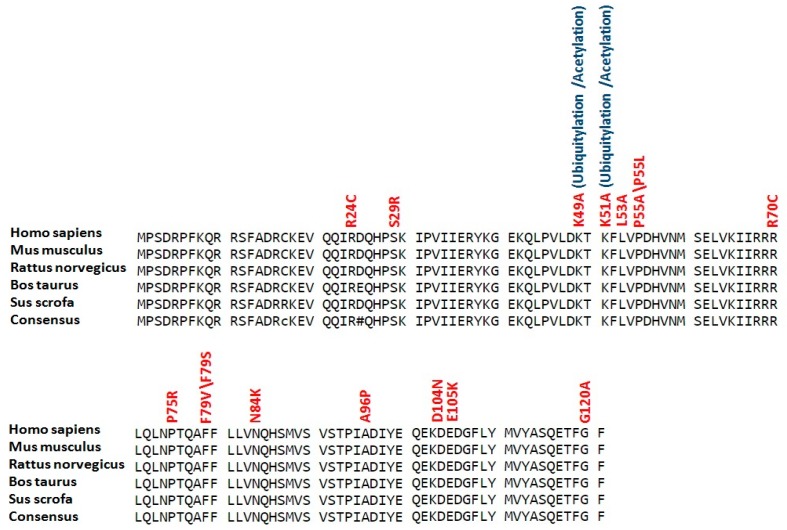
Conservation analysis of potential modified residues in LC3A protein. Sequences of LC3A proteins (human, mouse, rat, cattle and pig) were aligned using Multalin version 5.4.1 (http://multalin.toulouse.inra.fr/multalin/). High risk deleterious missense SNPs are mentioned in red color at respective positions while overlap with various types of posttranslational modifications are given in blue color.

**Figure 3:**
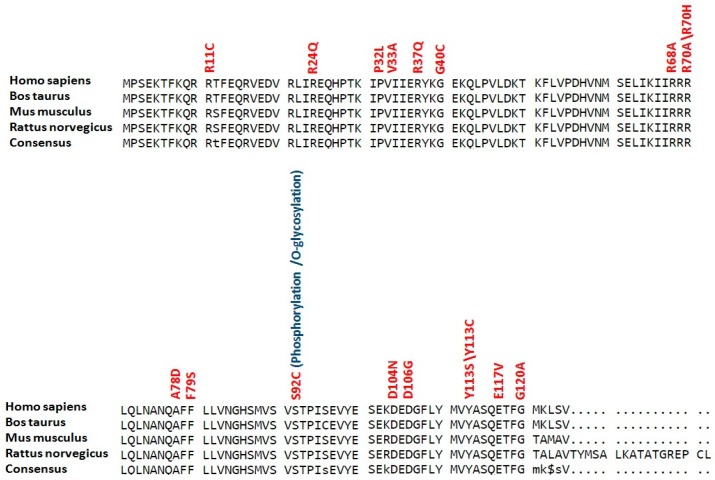
Conservation analysis of potential modified residues in LC3B protein. Sequences of LC3B proteins (human, mouse, rat and cattle) were aligned using Multalin version 5.4.1 (http://multalin.toulouse.inra.fr/multalin/). High risk deleterious missense SNPs are mentioned in red color at respective positions while overlap with various types of posttranslational modifications are given in blue color.

**Figure 4:**
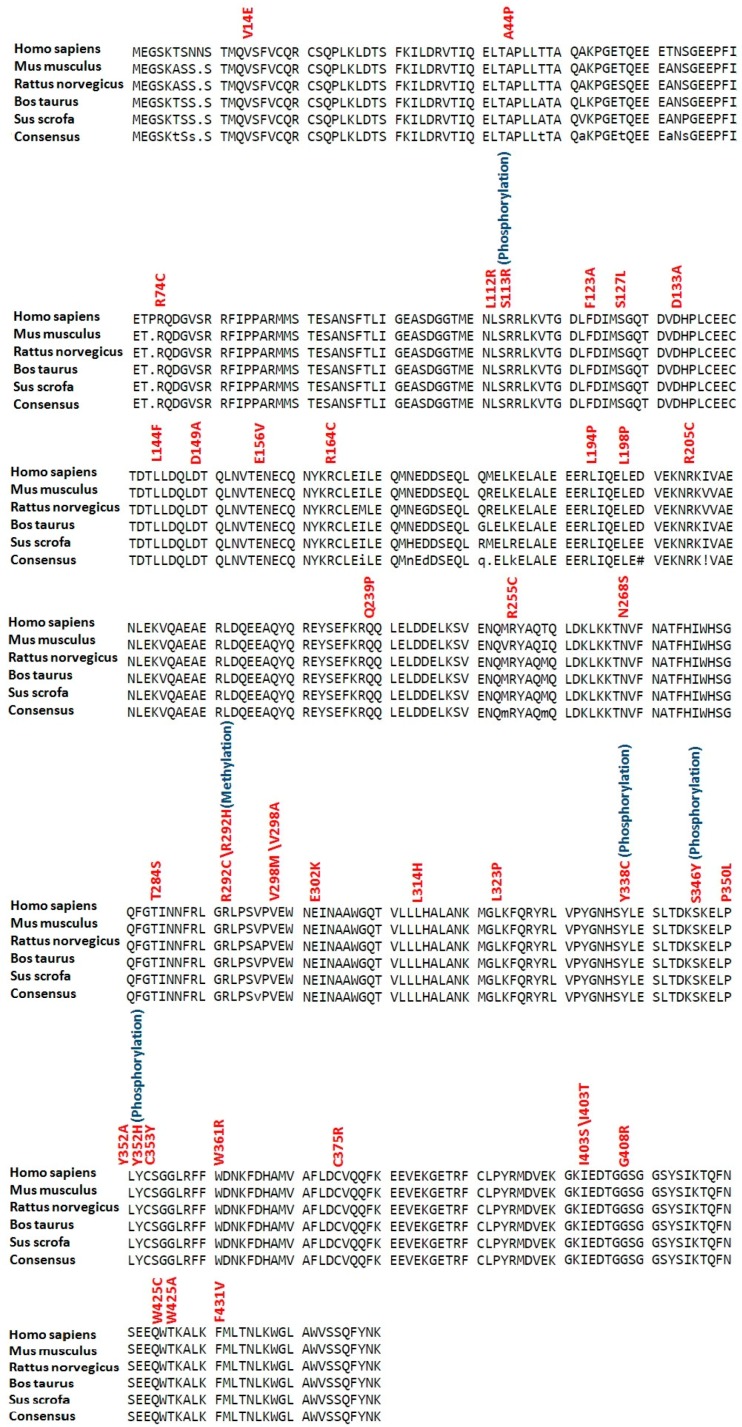
Conservation analysis of potential modified residues in BECN1 protein. Sequences of BECN1 proteins (human, mouse, rat, cattle and pig) were aligned using Multalin version 5.4.1 (http://multalin.toulouse.inra.fr/multalin/). High risk deleterious missense SNPs are mentioned in red color at respective positions while overlap with various types of posttranslational modifications are given in blue color.

**Figure 5:**
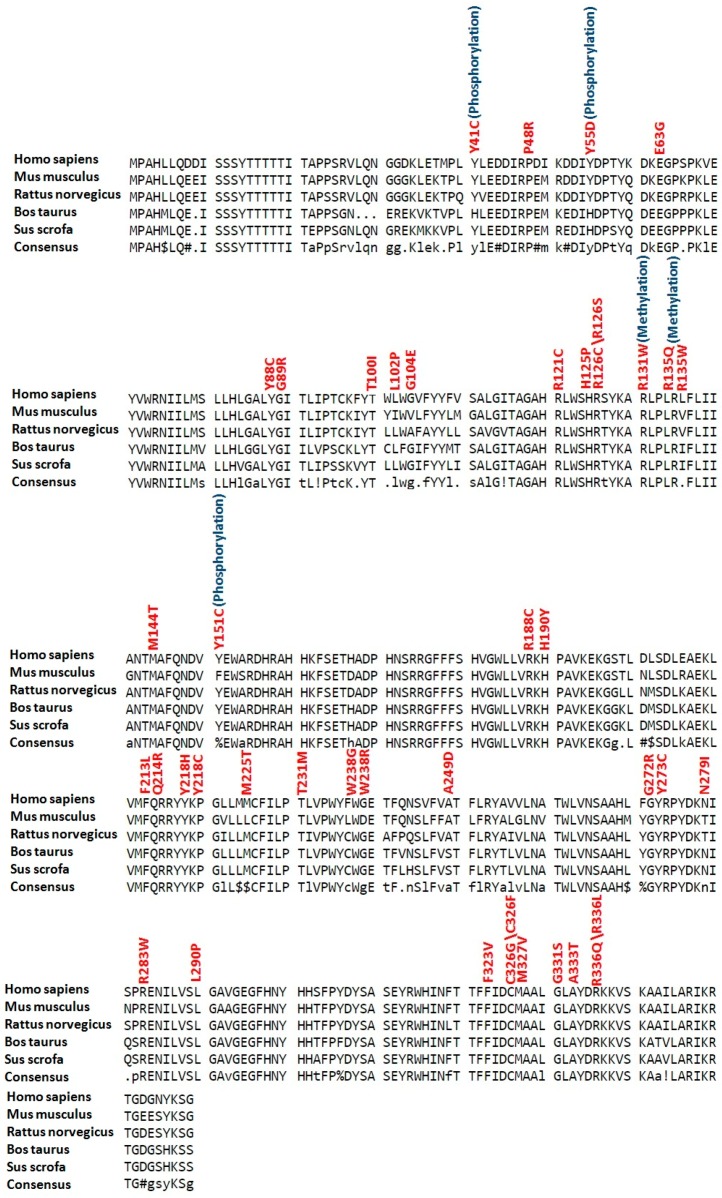
Conservation analysis of potential modified residues in SCD1 protein. Sequences of SCD1 proteins (human, mouse, rat, cattle and pig) were aligned using Multalin version 5.4.1 (http://multalin.toulouse.inra.fr/multalin/). High risk deleterious missense SNPs are mentioned in red color at respective positions while overlap with various types of posttranslational modifications are given in blue color.

Conservation analysis revealed that all residues with high risk SNPs in LC3A, LC3B and BECN1 were highly conserved (see Figure 2, Figure 3 and Figure 4). While in SCD1, 30 residues (P48, E63, Y88, G89, T100, R121, H125, R126, R131, R135, M144, Y151, R188, H190, F213, Q214, Y218, M225, T231, W238, G272, Y273, R283, L290, F323, C326, M327, G331, A333 and R336) with high risk SNPs were highly conserved (see Figure 5).

Further, the protein stability change upon point mutation was calculated using I-Mutant2.0, DUET and STRUM web-servers. Only highly deleterious missense SNPs were considered for the analysis. SNPs were considered as destabilizing in nature if two or more than two algorithms showed a decrease in stability upon mutation. In LC3A, one high-risk SNP (P75R) was found to be more stable than the wild-type (WT) LC3A. All high-risk missense SNPs in LC3B were found to be less stable than the WT LC3B and in BECN1 protein, 1 (A44P) out of 40 high-risk missense SNPs was predicted to be more stable than WT protein while in SCD1 protein, 5 (T100I, H125P, H190Y, N279I, R336L) out of 43 high-risk missense SNPs were predicted to be more stable than WT protein.

### 2.4. HCC Related Mutations by PinSnps and SNPEffect4.0 Tool

**Figure 6:**
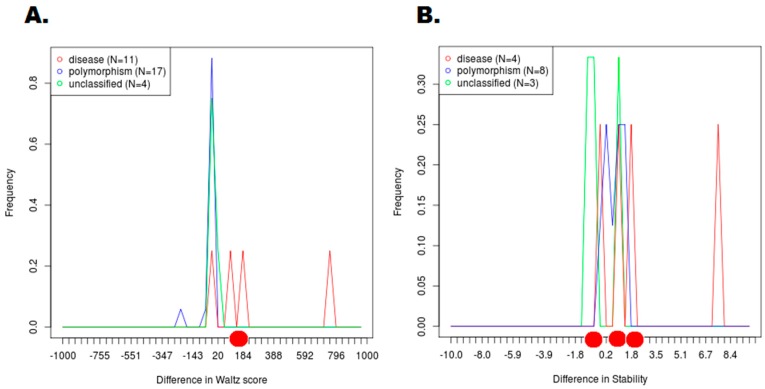
Representation of WALTZ and DDG scores for the mutations associated with HCC. (**A**) Difference in WALTZ scores with respect to its frequency of occurrence (red dot represents WALTZ score for LC3B Y113C); (**B**) Difference in stability scores with respect to its frequency of occurrence (red dot represents DDG scores for BECN1 I403T , SCD1 R126S and SCD1 Y218C).

In order to determine HCC associated SNPs in LC3A, LC3B, BECN1 and SCD1, PinSnps tool was used. Only highly damaging missense SNPs were analyzed to find association with HCC. A very interesting scenario was observed in the results retrieved from PinSnps tool. In LC3A, no mutation was found to be associated with HCC. In LC3B, 1 highly damaging missense SNP (Y113C) was found to be associated with HCC, while in BECN1, results showed that 1 highly deleterious missense SNP mutation i.e., I403T has a phenotype in HCC. In SCD1, R126S and Y218C (both highly deleterious missense SNPs) were found to be associated with HCC. SNPEffect4.0 results revealed significant fluctuations in scores of HCC associated highly damaging SNPs. The scores were observed to lie in the distribution range of HCC associated mutations (WALTZ score for LC3B Y113C and DDG scores for BECN1 I403T, SCD1 R126S and SCD1 Y218C) (see Figure 6). Other scores retrieved from SNPEffect4.0 tool did not revealed much significance.

### 2.5. Prediction of Functional Binding Sites

The 3D structures of LC3A and LC3B proteins were retrieved from protein data bank (PDB) with PDB IDs 4ZDV and 2ZJD respectively. For BECN1 and SCD1, top predicted models were selected on the basis of C-score. PROCHECK and ERRAT web servers were further used to verify the reliability of predicted models. The results of the predicted 3D BECN1 model showed 77.4% of residues in favored region, 19.0% in additional allowed region, 2.4% in generously allowed regions and just 1.2% in outlier region which highly indicates a good stereochemical quality of the predicted model (see Figure S2). Similarly, the results of the predicted 3D SCD1 model showed 73.4% of residues in favored region, 20.1% in additional allowed region, 4.4% in generously allowed regions and 2.2% in outlier region (see Figure S3). Moreover, ERRAT, a protein structure verification web server was used to verify the model on the basis of model building and refinement, and is extremely useful in making decisions about reliability of the model. ERRAT results showed that the overall quality factor for BECN1 model is 89.786%, whereas for SCD1 model, it is 80.627%, suggesting that the generated models are robust.

**Figure 7:**
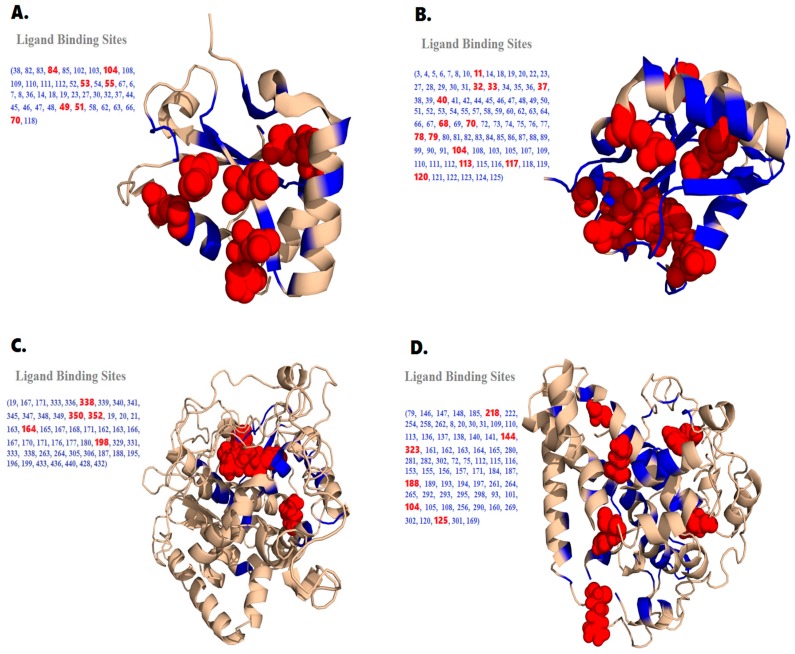
Human LC3A (PDB ID: 4ZDV) (**A**), LC3B (PDB ID: 2ZJD) (**B**), BECN1 (**C**) and SCD1 (**D**) in cartoon and a ball-and-stick representations. Ligand binding pockets were annotated by FTsite and COACH servers. Ligand binding sites coinciding with highly deleterious missense mutations are shown as ball-and-sticks in red color. Illustration was created using PYMOL.

FTSite tool, an energy based approach and COACH server were utilized to locate functional ligand binding sites on autophagy proteins by using PDB files as an input. In LC3A, 3 potential binding sites were identified using FTSite. 14 different amino acid residues were found to lie in the binding site 1 (Lys 8, Tyr 38, Leu 82, Val 83, Asn 84, Gln 85, Glu 102, Lys 103, Asp 104, Phe 108, Leu 109, Tyr 110, Met 111, Val 112). Second binding site contain nine residues (Pro 6, Phe 7, Lys 8, Ile 34, Ile35, Glu 36, Tyr 110, Met 111, Val 112), while third binding site contain nine residues (Ile35, Phe 52, Leu 53, Val 54, Pro 55, Leu 63, Ile 66, Ile 67, Arg 70). COACH server predicted 26 residues being potential sites involved in binding including 14, 18, 19, 23, 27, 30, 32, 37, 44, 45, 46, 47, 48, 49, 51, 52, 53, 54, 55, 58, 62, 63, 66, 70, 108, 118). Further analyses revealed that seven high risk missense SNPs were located in these binding sites (49, 51, 53, 55, 70, 84, and 104) (see Figure 7A).

In LC3B, 3 potential binding sites were identified using FTSite. For the binding site 1, 10 amino acid residues were found (Ile64, Arg 68, Phe79, Phe80, Leu81, Leu82, Met88, Val89, Ser90, Val91). Second binding site contain sixteen residues (Ser3, Glu4, Lys5, Thr6, Phe7, Arg10, Glu36, Arg37, Tyr38, Lys39, Gly40, Glu41, Lys42, Gly85, His86, Val112), while third binding site contain ten residues (Thr 6, Phe 7, Lys 8, Ile34, Ile35, Glu 36, Gly85, Tyr 110, Met111, Val 112). COACH server predicted 88 residues being potential sites involved in binding including 6, 7, 8, 10, 11, 14, 18, 19, 20, 22, 23, 27, 28, 29, 30, 31, 32, 33, 34, 35, 36, 37, 38, 39, 40, 41, 44, 45, 46, 47, 48, 49, 50, 51, 52, 53, 54, 55, 57, 58, 59, 60, 62, 63, 64, 66, 67, 69, 70, 72, 73, 74, 75, 76, 77, 78, 79, 80, 81, 82, 83, 84, 85, 86, 87, 89, 99, 104, 108, 103, 105, 107, 109, 110, 111, 112, 113, 115, 116, 117, 118, 119, 120, 121, 122, 123, 124, 125). Further analyses revealed that fifteen high risk missense SNPs were located in these binding sites (11, 32, 33, 37, 40, 68, 70, 78, 79, 104, 113, 117, 120) (see Figure 7B).

In BECN1, 3 potential binding sites were identified. For the binding site 1, 15 different amino acid residues were found to interact (Gln 19, Glu 167, Gln 171, Tyr 333, His 336, Tyr 338, Leu 339, Glu 340, Ser 341, Lys 345, Lys 347, Glu 348, Leu 349, Pro 350 and Tyr 352). Second binding site contain nine residues (Gln 19, Arg 20, Cys 21, Lys 163, Arg 164, Cys 165, Glu 167, Ile 168 and Gln 171), while third binding site contain fourteen residues (Tyr 162, Lys 163, Leu 166, Glu 167, Glu 170, Gln 171, Asp 176, Ser 177, Leu 180, Leu 198, Arg 329, Val 331, Tyr 333 and Tyr 338). COACH server predicted 14 residues being potential sites involved in binding including 263, 264, 305, 306, 187, 188, 195, 196, 199, 433, 436, 440, 428 and 432. Further analyses revealed that five high risk missense SNPs were located in these binding sites (164, 338, 350, 352 and 198) (see Figure 7C).

In SCD1, 3 potential binding sites were identified. Binding site 1 is comprised of 10 different amino acid residues (Met 79, Phe 146, Gln 147, Asn 148, Leu 185, Tyr 218, Leu 222, Tyr 254, Leu 258 and Trp 262). Second binding site contain fourteen residues (Asp 8, Ile 20, Asn30, Gly 31, Phe 109, Val 110, Leu 113, Leu 136, Phe 137, Leu 138, Ile 140, Ala 141, Met 144 and Phe 323), while third binding site contain nine residues (His 161, Lys 162, Phe 163, Ser 164, Glu 165, Ile 280, Ser 281, Pro 282 and His 302). COACH server predicted 47 residues being potential sites involved in binding including 72, 75, 112, 115, 116, 146, 147, 148, 153, 155, 156, 157, 171, 184, 185, 187, 188, 189, 193, 194, 197, 254, 258, 261, 262, 264, 265, 292, 293, 295, 298, 93, 101, 104, 105, 108, 109, 256, 290, 160, 269, 302, 120, 125, 161, 301 and 169. Further analyses revealed that six high risk missense SNPs were located in these binding sites (144, 323, 218, 188, 104 and 125) (see Figure 7D).

### 2.6. Phosphorylation and Kinase-Specific Phosphorylation Sites in LC3A, LC3B, BECN1 and SCD1

Three sites (Ser: 1, Thr: 1 and Tyr: 1) in LC3A revealed high potential for phosphorylation including Ser 12, Thr 93 and Tyr 99. Three Ser residues (Ser 90, Ser 92 and Ser 115), one Thr residue (Thr 50) and one Tyr residue (Tyr 110) showed probable phosphorylation potential. NetSurfP program was utilized to determine the surface accessibility of potential predicted Ser, Thr and Tyr residues of LC3A, LC3B, BECN1 and SCD1 for phosphorylation. Determining the surface availability of potential sites is of vital importance as residues buried in the core of protein would not be exposed to any kinase. Results revealed six residues Ser 12, Thr 50, Ser 90, Ser 92, Thr 93 and Ser 115 in LC3A to be accessible for phosphorylation by predicting these residues as exposed surfaces. Tyr 99 and Tyr 110 were predicted to be buried by NetSurfP server. YinOYang 1.2 server predicted 5 potential *O*-glycosylation sites including Ser 3, Ser 29, Ser 87, Ser 90 and Tyr 118. One potential YinOYang site was predicted in LC3A at residue position Ser 90.

Four sites (Ser: 1, Thr: 2 and Tyr: 1) in LC3B were identified to have high potential for phosphorylation unveiling Ser 101, Thr 6, Thr 93 and Tyr 99. Three Ser residues (Ser 90, Ser 92 and Ser 115), three Thr residues (Thr 12, Thr 29 and Thr 50) and one Tyr residue (Tyr 110) showed probable potential for phosphorylation. NetSurfP results revealed nine residues being surface exposed in LC3B including Ser 101, Thr 6, Thr 93, Ser 90, Ser 92, Ser 115, Thr 12, Thr 29 and Thr 50. Two residues Tyr 99 and Tyr 110 were predicted to be buried by NetSurfP server. YinOYang 1.2 server predicted 5 potential O-glycosylation sites including Ser 3, Thr 29, Ser 90 Ser 92 and Ser 124. Three potential YinOYang sites were predicted in LC3B at residue position Thr 29, Ser 90 and Ser 92.

Seventeen sites (Ser: 11, Thr: 3 and Tyr: 3) in BECN1 revealed high potential for phosphorylation unveiling Ser 30, Ser 64, Ser 79, Ser 90, Ser 113, Ser 177, Ser 249, Ser 337, Ser 346, Ser 414, Ser 421, Thr 57, Thr 72, Thr 259, Tyr 229, Tyr 352 and Tyr 413. Eight Ser residues (Ser 7, Ser 10, Ser 15, Ser 96, Ser 104, Ser 234, Ser 341, Ser 409), five Tyr residues (Tyr 162, Tyr 233, Tyr 256, Tyr 333, Tyr 338) and ten Thr residues (Thr 6, Thr 38, Thr 62, Thr 91, Thr 130, Thr 143, Thr 150, Thr 343, Thr 406, Thr 417) showed probable potential for phosphorylation. Results revealed thirty residues Ser 64, Ser 79, Ser 90, Ser 113, Ser 177, Ser 249, Ser 346, Ser 421, Thr 57, Thr 72, Thr 6, Thr 38, Thr 62, Thr 91, Thr 130, Thr 143, Thr 150, Thr 343, Thr 406, Ser 7, Ser 10, Ser 96, Ser 104, Ser 234, Ser 341, Ser 409, Tyr 256, Tyr 333, Tyr 338 and Thr 259 in BECN1 to be available for phosphorylation by predicting these residues as exposed. Ten residues including Ser 30, Ser 337, Ser 414, Tyr 229, Tyr 352, Tyr 162, Tyr 233, Thr 417, Ser 15 and Tyr 413 were predicted to be buried by NetSurfP server. YinOYang 1.2 server predicted 10 potential O-glycosylation sites including Thr 6, Thr 11, Ser 15, Ser 22, Ser 48, Ser 79, Thr 91, Thr 119, Ser 444 and Ser 445. Four potential YinOYang sites were predicted in BECN1 at residue positions Thr 6, Ser 15, Ser 79 and Thr 91.

Fifteen sites (Ser: 9, Thr: 1 and Tyr: 5) in SCD1 revealed high potential for phosphorylation unveiling Ser 13, Ser 66, Ser 124, Ser 164, Ser 173, Ser 198, Ser 203, Ser 281, Ser 340, Thr 58, Tyr 41, Tyr 55, Tyr 59, Tyr 71 and Tyr 151. Five Ser residues (Ser 11, Ser 12, Ser 127, Ser 309, Ser 311), five Thr residues (Thr 17, Thr 18, Thr 19, Thr 166, Thr 199) and seven Tyr residues (Tyr 14, Tyr 217, Tyr 254, Tyr 306, Tyr 308, Tyr 313, Tyr 356) showed probable potential for phosphorylation. Results revealed fifteen residues as surface exposed including Ser 12, Ser 66, Ser 164, Ser 198, Ser 203, Ser 281, Ser 340, Thr 58, Tyr 41, Tyr 55, Thr 19, Thr 166, Thr 199, Tyr 356 and Tyr 59 in SCD1. Sixteen residues including Ser 124, Ser 173, Tyr 71, Ser 11, Ser 127, Thr 17, Thr 18, Ser 309, Ser 311, Tyr 14, Tyr 217, Tyr 254, Tyr 306, Tyr 308, Tyr 313 and Tyr 151 were predicted to be buried by NetSurfP server. YinOYang 1.2 server predicted 9 potential O-glycosylation sites including Ser 11, Thr 18, Thr 19, Thr 21, Ser 25, Ser 173, Ser 340, Thr 351 and Ser 358. Five potential YinOYang sites were predicted in SCD1 at residue positions Ser 11, Thr 18, Thr 19, Ser 173 and Ser 340.

KinasePhos 2.0 and GPS 3.0 servers were used for the prediction of kinase-specific phosphorylation sites in human LC3A, LC3B, BECN1 and SCD1. Several kinases play fundamental role in the initiation and execution of autophagy. Mitogen activated protein kinases (MAPKs) are serine/threonine kinases that regulate autophagy in response to various stimuli [29]. The best-studied MAPKs are ERK, JNK and p38 which are activated in response to various stresses and proliferative signals. In BECN1, Ser at position 90 revealed high potential for phosphorylation. Previously, it is known that this phosphorylation site is essential for the tumor suppressor function of BECN1. The authors revealed that members of the p38 MAPK signaling pathway including MAPKAPK2 (MK2) and MAPKAPK3 (MK3) positively regulate starvation-induced autophagy by phosphorylating BECN1 at Ser 90 [30]. Advanced computational algorithms used in this study revealed Ser 90 as a potential phosphorylation site for MAPKAPK5 (MK5) also, including MK2 and MK3, warranting experimental studies in this regard. Current findings further revealed Thr 93 in LC3A as a potential phosphorylation site for ERK, p38/MAPK12 and MAPK14 whereas, in LC3B, Thr 93 as a potential phosphorylation site for ERK, JNK, p38/MAPK12 and MAPK14. In BECN1, Thr 6 and Ser 7 revealed high potential for MAPK13, Thr 72 for MAPK11, ERK4, JNK1 and Ser 79 for ERK3, Ser 409 for MAPK11, MAPK14 and Thr 417 revealed high potential for phosphorylation by MAPK13. In SCD1, Ser 66 revealed high potential for MAPK12, MAPK14, ERK3 and JNK3 whereas Ser 281 revealed high potential for p38 MAPK, ERK4 and JNK3. The mammalian target of rapamycin (mTOR), an energy-sensing kinase, is considered the master regulator of autophagy [31]. During prolonged starvation, degradation of autolysosomal products results in reactivation of mTOR [32]. In SCD1, Thr 17 was predicted to have probable potential for phosphorylation by mTOR whereas in LC3A, Thr 93 was predicted to have high potential for phosphorylation by mTOR. Ribosomal protein S6 kinase beta-1 (RPS6KB1), a serine/threonine kinase acts downstream of PIP3 in the PI3 kinase pathway [33]. RPS6KB1 is known to have a positive regulatory role in autophagy [34]. In BECN1, Ser at positions 64, 10, and 234 were predicted to have high potential for phosphorylation by RPS6KB1. Autophagy is promoted by AMP activated protein kinase (AMPK), a key energy sensor. It regulates cellular metabolism to maintain energy homeostasis [35]. Under glucose starvation, AMPK activates autophagy protein Ulk1 through phosphorylation of Ser 317 and Ser 777. Under nutrient sufficiency, high mTOR activity disrupts the interaction between Ulk1 and AMPK by phosphorylating Ulk1 at Ser 757 [36]. Current results also revealed that in LC3A and LC3B, Ser at positions 92 and 115 whereas in BECN1, Thr at positions 57, 259 and Ser at positions 104 and 234 revealed high portential for phosphorylation by AMPK.

### 2.7. Normal Mode Analysis of Highly Deleterious HCC-Associated SNPs

iMODS provides a user-friendly interface for enhanced normal mode analysis methodology. The detailed analysis includes profiles of mobility (B-factors), eigenvalues, covariance map, deformability and linking matrix. The corresponding eigenvalues signifies the total mean square fluctuations. The eigenvalue is directly related to the energy required to deform the structure and represents the motion stiffness. The lower the eigenvalue, the easier the deformation. The results of iMOD server revealed that the eigenvalue of all four HCC-associated SNPs were lower as compared to WT proteins which indicates different behavior of mutant and wild-type proteins (see Figures S4–S7).

### 2.8. Ubiquitylation Sites in LC3A, LC3B, BECN1 and SCD1

In LC3A and LC3B, lysine residues at position 42, 49 and 51 were predicted to have high potential for ubiquitylation. In BECN1, lysine residues at position 5, 53 and 237 were predicted to have high potential for ubiquitylation. In SCD1, lysine residues at position 162, 196, 209, 341 and 357 were predicted to have high potential for ubiquitylation.

### 2.9. Palmitoylation and Methylation Sites in LC3A, LC3B, BECN1 and SCD1

In LC3A, Cys residue at position 17 was found to have high potential for palmitoylation. No potential palmitoylation site was predicted in LC3B. In BECN1, Cys residue at position 18 and 21 were found to have high potential for palmitoylation. In SCD1, Cys residue at position 226 was found to have high potential for palmitoylation.

In LC3A and LC3B, R residue at position 10 was found to have high potential for methylation. In BECN1, R residues at position 36, 80, 87, 292, 329, 358 and 395 were predicted to have high potential for methylation. In SCD1, R residues at position 131, 135, 155, 175, 314 and 347 were predicted to have high potential for methylation.

### 2.10. Prediction of Acetylating Sites in LC3A, LC3B, BECN1 and SCD1

In LC3A, residues at position 8, 42, 51 and 49 were predicted to have high potential for acetylation. In LC3B, residues at position 5, 8, 42, 51, 49 and 122 were predicted to have high potential for acetylation. In BECN1, residues at position 5, 26, 53, 237, 266, 324, 345, 416 and 450 were predicted to have high potential for acetylation. In SCD1, residues at position 62, 68, 129, 162, 194, 196, 209, 278, 337, 338, 341, 349, 357 were predicted to have high potential for acetylation.

### 2.11. Sumoylation Sites in LC3A, LC3B, BECN1 and SCD1

No sumoylation site was predicted in LC3A and LC3B. In BECN1, residue at position 380 was predicted to be potential SUMOylation site with high confidence level. No other potential SUMOylation site was predicted in BECN1 by more than one algorithm. In SCD1, residues at position 51 and 68 were predicted to have high potential for sumoylation.

### 2.12. Analysis of Overlap between High Risk SNPs and Potential PTM Sites

PTMs play key roles in various cellular processes and their dysfunction due to SNPs could result in human diseases [18]. For example, PTN11_HUMAN was shown to carry 23 PTMs with 55 functional associations with 4 variations occurring on phosphorylation sites (T2I, Y62D, Y63C, and Y279C) and 1 on acetylation site of (Y279S). The mutations on Y279 are associated with “human LEOPARD syndrome 1 and the mutations on the remaining sites are associated with “human Noonan syndrome 1. Analysis of our results showed that 2 highly damaging SNPs in LC3A, 1 in LC3B, 6 in BECN1 and 6 in SCD1 coincide with potential PTM sites.

## 3. Discussion

SNPs are commonly found across the genome and play an important role in diversifying protein function. Amino acid variations as a result of SNPs may cause changes in protein stability, disrupt salt bridges or hydrogen bonding thus affecting protein dynamics. It may also disrupt the binding site and affect protein interaction by altering the specificity of the protein, blocking the active site or affecting the binding affinity. Identifying highly deleterious SNPs or those responsible for a specific phenotype and characterizing these deleterious mutations can be a complex and intricate task in large-scale analyses which is of a major concern and requires testing thousands of SNPs in the respective genes. The ability to set apart deleterious SNPs from neutral ones using computational approaches could considerably help in targeting the disease related mutations. Therefore, there is an obvious need for in silico analysis. In the current study, we prioritized highly deleterious mutations in autophagy related proteins as well as HCC-associated missense mutations which require further experimental studies to confirm their damaging effect.

LC3A being indispensible for basal homeostasis and cellular recycling process of autophagy is regulated by several PTMs [37]. Seven highly damaging missense SNPs in LC3A were prioritized for further analysis including N84K, D104N, L53A, P55A, K49A, K51A and R70C. Suzuki et al. observed significant reduction in LC3A positive puncta formation in cultured cells after introducing mutation of Lys49. They reported that hydrophobic pockets of LC3A including residues Ile23, Lys49, Lys51, Phe52, Leu53, Ile66, and Phe108 interact with Phe444 of the ATG13 LIR and by hydrogen bonding between Lys51 and Leu53 of LC3A and Val445 and Ile446 of the ATG13 LIR. Using biosensor experiments, they observed increased relative binding ability of LC3A^K49A^ to ATG13 LIR [38]. In our study, K49A mutation which is predicted to be highly damaging SNP in LC3A also coincide with highly potential ubiquitylation and acetylation site revealing its significance. Mutations in evolutionary conserved acetylation and ubiquitination sites have been previously reported to be enriched in various tumors [39]. Future studies to unveil the importance of ubiquitylation and acetylation at this site and to explore the impact of K49A mutant on ubiquitylation and acetylation at this site will help to better understand the mechanism of autophagy during pathological conditions. Suzuki et al. investigated side-chain structural arrangements in ATG13-LC3A structures at a resolution of 1.77 Å. They observed that in ATG13-LC3A complex structure, residues Glu19, His27, Lys30, and Pro55 of LC3A are involved in the interaction with ATG13^436–447^. Upon binding, these residues move slightly to reorganize except for Lys49 which undergoes a large rotamer rearrangement [38]. As pro55 has been shown to be important residue for interaction with ATG13 LIR, therefore SNP at this site can be highly damaging for this interaction.

Fifteen highly damaging missense SNPs in LC3B were prioritized for further analysis including R11C, P32L, V33A, R37Q, G40C, R68A, R70A, R70H, A78D, F79S, D104N, Y113S, Y113C, E117V, G120A. Previously, R68 was found to be an important residue being necessarily required for the interaction between ATG8 family proteins and ATG4B. During autophagy process, R68 also plays significant role in C-terminal cleavage efficiency of ATG8 proteins. The authors found that its mutation severely decreased the cleavage and affected the autophagic localization and autophagic flux [40]. Results of the current study found out R68A as highly deleterious missense SNP. The absence of R68 significantly affected internal interaction in LC3 by altering the salt bridge between R70 and D48. Furthermore, the absence of R68 leads to diminution of one rotation in the α3 helix, a central factor in stabilizing the loops L1 and L2 interactions as well as by maintaining the ubiquitin fold structure. LC3-interacting region (LIR) motif along with N-terminal arm (basic residues) and ubiquitin-like (Ubl) domain of LC3B (R10, R11, K49 and K50) have been previously shown to be important for its interaction with p62 [41]. Previously, residues Leu53 and Arg70 on hydrophobic surface in LC3B have been shown to be required for efficient binding with many LC3-interacting proteins. The authors reported that L53 altered the stoichiometry of LC3B complexes. Mutations in L53A and R70A on LC3B′s hydrophobic protein interacting surface altered binding to proteins involved in various activities including lipid modification and autophagy substrate specificity, highlighting the significance of these residues for engaging other proteins in dynamic binding [42]. Results of current study revealed that missense mutation Y113C is phenotypically associated with HCC. The mutant residue (Cys) is smaller and more hydrophobic than the WT residue (Tyr) ultimately affecting hydrogen bond formation. The WT residue forms a hydrogen bond with proline at position 45. Loss of hydrogen bonds in the core of the protein caused by mutant residue will also disturb correct folding of the protein (see Table S2). Substitution of a large side chain with a small one can be highly damaging for some protein structures. Prompt removal of unwanted cells by apoptosis and autophagy is crucial for the maintenance of liver health. Indeed, impaired and insufficient autophagy has been associated with the development and progression of HCC. Previously, Chen et al. reported that Y113 lies in caveolin-1 (Cav1) binding motif (^108^FLYMVYASQETF^119^) of LC3B and Y113A mutation (aromatic to non-aromatic substitution), significantly altered the basal LC3B-Cav1 interaction. This mutation resulted in reduced apoptosis than the WT LC3B construct revealing an important role of this residue in apoptosis. The reduced apoptosis of LC3B Y113A mutant as compared to WT LC3B was because of noticeable increase in Cav1-Fas interaction and resultant reduced activation of the extrinsic apoptotic pathway [43]. HCC is a hypoxic solid tumor. Cav-1 expression has been closely related to the aggressiveness of the HCC [44]. The study conducted by Lee et al. showed that under normoxia, Cav1 and LC3B are localized in caveolae, while hypoxia induced the translocation of these proteins from the membrane to the cytoplasm [45]. Chen et al. discovered that Cav1 is crucial for mediating LC3B-Fas complex which is localized in lipid rafts in plasma membrane. Caveolin scaffolding domain (CSD) in Cav-1 is the region that binds protein partners. CSD of Cav1 binds canonical Cav1-binding motifs (CBMs) on LC3B, while Fas binds to the palmitoylation domains of Cav1. The proapoptotic function of LC3B requires its direct interaction with Cav1, since site-directed mutagenesis on LC3B at its Cav-1 binding motif disrupted its apoptotic function. Therefore, we speculated that Y113C mutation in LC3B will disrupt its binding with Cav1 and will impair its apoptotic function, ultimately reducing apoptosis that could lead towards the progression of HCC.

Mitophagy is mitochondria selective autophagy which plays a pivotal role in the elimination of the damaged mitochondria. The defects in mitophagy are associated with a wide spectrum of human diseases. LC3B has also been known to contain cardiolipin-binding sites which are important for mitophagy [46]. Cardiolipin is an inner unique dimeric phospholipid mitochondrial membrane. Under mild mitochondrial damage, cardiolipin is redistributed to the outer mitochondrial membrane and serves as a recognition signal for dysfunctional mitochondria, which are rapidly sequestered by autophagosomes. The study conducted by Chu et al. revealed the involvement of LC3B residues K5, R10 and R11 in the initial interactions with the cardiolipin-containing bilayer, with recruitment of residues R68-R70 and K49. Mutation of these residues significantly altered LC3B’s participation in mitophagy unveiling the significance of these sites. Results of the current study found out R11C, R68A and R70A as highly deleterious missense SNP unveiling their significance.

Ten highly damaging missense SNPs in BECN1 were prioritized for further analysis including S113R, R292C, R292H, S346Y, I403T, Y338C, P350L, Y352H, L198P and R164C. Lee et al. reported that in starvation condition, cell death activity was slightly reduced in P350R and R389C mutants compared to the WT BECN1 revealing some role of theses residues in apoptosis. The results also proposed cell death activity of C-terminal portion of the BECN1 protein [47]. Interaction between EGFR and autophagy protein BECN1 has been previously observed, leading to BECN1 phosphorylation, decreased BECN1-associated kinase activity and enhanced binding to inhibitors, while enhanced tumor growth, tumor dedifferentiation and reduced autophagy was observed in BECN1 mutants. The authors identified Y352 being the substrates of EGFR-mediated Beclin 1 tyrosine phosphorylation. EGFR tyrosine kinase phosphorylates BECN1 at residue Y352, which in turn inhibited autophagic activity by enhancing Beclin 1 interaction with Bcl-2 [48]. Y352H is predicted to be highly damaging SNP in our study. The Tyr residue at position 352 is also predicted to have a very high potential for phosphorylation in the current study. In the context of above studies, mutation at this site will significantly alter the tyrosine phosphorylation site ultimately reducing autophagy and enhancing tumor progression. Ser 113 is located within the BH3 domain of BECN1 [49]. Furuya et al. reported that the most highly conserved region of BECN1 spanning from amino acids 244-337 is indispensable for autophagy, Vps34 binding and tumor suppressor function [50]. Two highly damaging SNPs R292C and R292H lies in this region suggesting possible damaging role of theses SNPs in BECN1-Vps34 interaction. L198P lies in coiled-coiled domain of BECN1 (residue 174–266) which provides an interaction podium for ATG14L/UVRAG complexes to modulate VPS34 activity [50]. R164C lies in the partially disordered flexible helical domain (FHD) of BECN1 including residues 141–171. The FHD may undergo a disorder-to-helix transition for the starvation-induced up-regulation of autophagy as well as for binding to appropriate protein partners. The authors reported that FHD residues play an important role in starvation-induced autophagy [51]. Mice with mono-allelic loss of BECN1 or bi-allelic deletion of ATG5 in liver have been shown to be more prone to liver tumors [52]. Autophagy deficiency may promote initiation of benign liver tumors by inducing chronic tissue damage. On the other hand, autophagy may be needed for progression to more aggressive disease. Therefore, it will be of interest to examine the mutational status of autophagy genes in human hepatomas. This will test if autophagy defects will promote the genesis of hepatomas or they will limit tumor progression to benign disease [52]. BECN1 residues (246–339) were also found to be important residues for its association with NACHT, LRR and PYD domains-containing protein 4 (NLRP4). This interaction negatively regulates autophagy [53]. Highly deleterious mutations including R292C, R292H and Y338C might be severely damaging for BECN1 association with NLRP4. BECN1 is not only required for the formation of autophagosomes but also been previously found to play an important role in the initiation of mitophagy [54]. Hepatitis C virus, a major risk factor of HCC, causes mitochondrial injury and oxidative stress. Impaired mitochondria are selectively eliminated through mitophagy. The C-terminal part of BECN1 (residues 136–450) has a highly conserved nature, and is sometimes known as evolutionarily-conserved domain (ECD) [51]. BECN1 residue I403 lies in ECD. ECD comprises three structural repeats (1, 2, and 3) containing amino acids 269–323, 324–380, and 387–444, respectively [55]. BECN1 ECD, being a membrane-binding domain exhibits a strong preference for cardiolipin enriched lipid membranes. Cardiolipin is an inner unique dimeric phospholipid mitochondrial membrane. Under mild mitochondrial damage, cardiolipin is redistributed to the outer mitochondrial membrane and serves as a recognition signal for dysfunctional mitochondria, which are rapidly sequestered by autophagosomes. I403T mutant BECN1 might associate inadequately with lipid membrane and finally affect mitophagy process, ultimately progressing towards HCC. Moreover, in case of I403T mutation, the mutant residue (Thr) is smaller and less hydrophobic than the WT residue (Ile). I403 is buried in the core of a domain. The mutant residue might perturb the core structure of this domain and can create an empty space in the core of the protein (see Table S3).

Thirteen highly damaging missense SNPs in SCD1 were prioritized for further analysis including Y41C, Y55D, R126S, R131W, R135Q, R135W, Y151C, R188C, G104E, H125P, Y218H, Y218C, M144T and F323V. H125P lies in histidine box-1. Cytoplasmic cap domain of SCD1, which includes three histidines at positions 120, 125 and 269, facilitates in both metal binding and substrate recognition [56]. Therefore, mutation of H125 might affect the metal binding ability of SCD1. R188 have been previously reported to be an important reside residing in active binding site [56]. The life cycle of hepatitis C virus (HCV) which is the main risk factor for HCC is tightly regulated by the host cell metabolism. SCD1, a liver specific enzyme, is known to be associated with HCV replication complex and regulates its replication via its enzymatic activity [57]. Highly deleterious mutations in SCD1 can be damaging to various processes regulated by this protein. In Y218C mutation, the mutant residue is smaller and more hydrophobic than the WT residue which may affect the hydrogen bond formation with glutamine at position 147 (see Table S4). The amino acid cysteine is small in size. In some protein structural contexts, substitution of a large side chain for a small one can be highly damaging. R126 lies close to histidine box 1 (positions 120–125) therefore its mutation might seriously alter the functions performed by histidine box 1. Moreover, the positively charged WT residue (Arg) is replaced by a neutral residue (Ser) which will alter the ionic interaction made by the WT residue. The WT residue forms a hydrogen bond with aspartic acid at position 204 and with glutamic acid at position 208. The difference in hydrophobicity will affect hydrogen bond formation. The mutant residue will also disturb the formation of salt bridges made by WT residue with aspartic acid at position 204 and glutamic acid at position 208.

Predicting the consequences of amino acid variations on protein stability remains one of the most promising setbacks in protein science. Using computational approaches to filter out the most likely pathological variants from the huge pool of SNP datasets will be more fruitful in the years ahead. In our analysis, we identified the most deleterious mutation in LC3A, LC3B, BECN1 and SCD1 based on various bioinformatic tools. High risk missense SNPs were screened for their deleterious impact on protein function based on these tools. Our study shows that SNP analysis and scrutinization could be a supreme platform for identifying variants that leads to various diseases. Hence, the in silico analysis we performed proved to be both practical and valuable for thorough understanding of various diseases, thereby greatly facilitating valuable resource for the pharmacogenmoics approach.

## 4. Materials and Methods

### 4.1. Protein Sequence Datasets

With an objective to predict and analyze various SNPs and PTMs, FASTA sequences of human LC3A, LC3B, BECN1 and SCD1 were retrieved from SWISSPROT sequence database, with entry names MLP3A_HUMAN, MLP3B_HUMAN, BECN1_HUMAN, ACOD_HUMAN and primary accession numbers Q9H492, Q9GZQ8, Q14457 and O00767 respectively. Sequence homologs were aligned to analyze the level of evolutionary conservation. The accession numbers for selected sequences for LC3A are Q9H492 (Human), Q2HJ23 (Bovine), D2KQR0 (Pig), Q91VR7 (Mouse), Q6XVN8 (Rat), for LC3B; Q9GZQ8 (Human), O41515 (Bovine), Q62625 (Mouse), Q62625 (Rat) ; for BECN1; Q14457 (Human), Q4A1L4 (Bovine), Q4A1L5 (Pig), O88597 (Mouse), Q91XJ1 (Rat) and for SCD1; O00767 (Human), Q9TT94 (Bovine), Q6RWA7 (Pig), P13516 (Mouse), P07308 (Rat). Multalin (http://multalin.toulouse.inra.fr/multalin/) [58] was used for the alignment of LC3A, LC3B, BECN1 and SCD1 sequences in order to get the conservation pattern of predicted potential sites.

### 4.2. Retrieval of Missense SNP Datasets

Polymorphism data for *LC3A*, *LC3B*, *BECN1* and *SCD1* genes were retrieved from NCBI dbSNP database (https://www.ncbi.nlm.nih.gov/SNP/) [59] using gene IDs (*LC3A* “84557”, *LC3B* “81631”, *BECN1* “8678”, *SCD1* “6319”) and the UniProt database (http://www.uniprot.org). NCBI dbSNP is a public resource for a broad collection of all identified genetic variations within and across different species. It is an online resource implemented to aid biology researchers and is part of National Center for Biotechnology Information (NCBI)’s search in association with the National Human Genome Research Institute (NHGRI) and retrieval system Entrez.

### 4.3. Analysis of Functional Consequences of Missense SNPs

Missense SNPs analysis was performed using four tools nsSNP Analyzer [60], PROVEAN (Protein Variation Effect Analyzer) [61], SNPs & GO [62] and PMUT [63] which characterizes missense SNPs as neutral or damaging to structure and function. SNPs predicted as functional or damaging by at least three servers were considered damaging. nsSNP Analyzer, a web-based server uses Random Forest method and integrates multiple sequences alignment along with protein structure analysis to predict the phenotypic effect of SNPs. PROVEAN web tool uses an alignment-based scoring approach to predict the functional consequences of substitutions. In PROVEAN, a threshold of −2.5 was used (a score of ≤−2.5 were considered “deleterious” while a score >−2.5 were considered “neutral”). PMUT uses a neural network based approach to predict disease-associated mutations. SNPs & GO algorithm has been considered highly accurate, which uses protein GO annotation information to predict whether a particular mutation is disease-related or not [64].

### 4.4. Analysis of Protein Stability Changes Due to High Risk Missense SNPs

Predicting the impact of missense mutations on the stability of protein is necessary to gain an insight into structure-function relationship of the protein and human disease diagnosis. To quantitatively predict the change in protein stability due to high risk missense mutations, I-Mutant version 2.0 [65], DUET [66] and STRUM [67] web servers were used. As an input in I-Mutant and STRUM servers, FASTA sequences of LC3A, LC3B, BECN1 and SCD1 retrieved from UniProt were used. PDB files of 3D structures were used as an input in DUET web server. Prediction of energy change can be performed using either protein structure and sequence information. Stability change of the mutated protein structure was calculated from Gibbs free energy change (DDG) using the following formula:

DDG: DG (mutant Protein) − DG (wild-type) in Kcal/mol(1)

I-Mutant predicts the sign of decrease or increase in DG with Reliability Index (RI). RI = 0 indicates lowest reliability and RI = 10 indicates highest reliability. The value of DDG < 0 indicates decrease in protein stability and value > 0 indicates increase in protein stability. During prediction of energy change, the pH was set at 7 and temperature was set at 25 °C for all SNP submissions. The discrimination among deleterious or functionally effective SNPs from the neutral ones is based on the notion that protein stability perturbation should be above a certain threshold DDG (±1 kcal/mol) for it to be functionally important.

DUET adopts an integrated computational approach that combines a machine learning algorithm (mCSM) and a statistical potential energy function (SDM) in a non-linear way and analyzes the results using Support Vector Machines (SVM). It uses 3D protein structures as an input to quantitatively predict the effects of mutation induced changes on protein stability.

STRUM, a machine learning-based stability change predictor, uses a gradient boosting regression approach. It combines various physics-based and knowledge-based energy functions, which helps enhance the robustness and accuracy of the method and make it applicable to various protein sequences, including those without experimental structures.

### 4.5. Analysis of Structural Specificity of Functional SNPs

To study the structural effects of high risk mutations in LC3A, LC3B, BECN1 and SCD1 proteins, Project “Have your Protein Explained” HOPE (http://www.cmbi.ru.nl/hope/home), an easy-to-use unique web tool, that analyses the structural effects of a point mutations, was used. To simulate structural features of mutations on native protein molecule, Project HOPE uses 3D structures of the proteins that are available in UniProt database. The web server utilizes FASTA, BLAST, UniProt, PDB, DSSP, HSSP, YASARA, ClustalW, and DAS server to make its prediction on protein structure. The HOPE server predicts structural variation between native protein and the variant models and can also build homology models independently (see Tables S1–S4).

### 4.6. Prediction of Phenotypic Consequence for Deleterious Missense SNPs

The phenotypic consequences of deleterious missense SNPs were predicted using PinSnps (http://fraternalilab.kcl.ac.uk/PinSnps/) [68], a tool that integrates 2587 genetic disorder-related SNPs and 587873 cancer-related variant. It is considered to be one of the largest collections of variants mapped onto 3D coordinates. This computational pipeline readily performs data analyses of PPI networks by using genetic and functional information mapped onto protein structures. UniProt accession numbers were used as an input to determine the association of high risk missense SNPs to determine the clinical phenotype data. SNPeffect4.0 web tool [69] was also used to detect the changes in LIMBO, TANGO, WALTZ and DDG scores for the given mutation. Scores lying in particular distribution range can be used to relate to particular cancer associated mutations. SNPeffect4.0 tool can search all variants of the selected cancer type and retrieve disease associated SNPs.

### 4.7. Prediction of Ligand Binding Sites in LC3A, LC3B, BECN1 and SCD1 Proteins

In order to annotate protein function and predict the impact of SNPs on structural level in LC3A, LC3B, BECN1 and SCD1 proteins, 3D structures are of vital importance. Iterative Threading ASSEmbly Refinement (I-TASSER) server [70] was used for the ab-initio structure prediction of BECN1 and SCD1. I-TASSER uses replica-exchanged Monte Carlo simulations to generate full length model of proteins from structural alignments. Low temperature decoys generated during the simulations were clustered by SPICKER to pinpoint low free-energy states. Top five cluster centroids were then used to generate full length models. C-scores (−5 to 2) reflects the quality of prediction models. The overall stereochemical properties of the I-TASSER predicted models were further assessed by Psi/Phi Ramachandran plot analysis using PROCHECK interactive server. Moreover, a protein structure verification web server, ERRAT [71] was also used to check the stereochemical parameters, the correctness of the overall fold/structure and errors over localized regions. ERRAT is an extremely valuable tool in making decisions about reliability of the model. To find whether these identified high risk SNPs are present in the protein binding regions of LC3A, LC3B, BECN1 and SCD1, we performed binding site prediction. This was achieved by COACH and FTSite servers, highly rated bioinformatic softwares for protein–ligand docking (COACH, http://zhanglab.umich.edu/COACH/) [72] (FTSite, http://ftsite.bu.edu/) [73]. COACH combines the use of the state-of-art tools (COFACTOR, FINDSITE, TM-SITE, S-SITE and ConCavity) to predict protein–ligand binding. First, the BECN1 and SCD1 primary sequences were provided as input to generate 3D structures using I-TASSER. I-TASSER feeds the 3D structure into the COACH pipeline for the prediction of ligand-binding site. COACH utilizes a semi-manually curated database “BioLiP” that houses data on known proteins with their specific ligands. The predicted ligands with their binding sites on the protein were selected based on their scoring values. FTSite tool is capable of predicting protein-ligand binding sites with high experimental precision of 94%.

### 4.8. Normal Mode Analysis

Performing simulations of appropriate time scales are fundamental for understanding the structure-function correlations. Coarse grain techniques such as normal mode analysis are a powerful computational method for studying large-amplitude molecular deformational motions and require relatively much less resources as compared to molecular dynamic simulations which can be very demanding in terms of resources. Normal mode analysis was performed via iMod server (iMODS) (http://imods.chaconlab.org) [74] using the basic interface with default values for all the parameters. Only highly deleterious HCC-associated missense SNPs were considered for the analysis.

### 4.9. Prediction of Post-Translational Modification Sites

#### 4.9.1. Prediction of Potential Phosphorylation and Kinase-Specific Phosphorylation Sites in LC3A, LC3B, BECN1 and SCD1

Phosphorylation is the addition of a phosphate group to a Ser, tyrosine (Tyr) or threonine (Thr) and is often considered a key event in response to cellular stimuli. An artificial neural network based program, Netphos 2.0 [75] was used for predicting the phosphorylation probability of human LC3A, LC3B, BECN1 and SCD1 for each Thr, Ser and Tyr residues. A minimum threshold value of 0.5 was chosen. KinasePhos 2.0 [76] and GPS 3.0 [77] servers were used for the prediction of kinase-specific phosphorylation sites in human LC3A, LC3B, BECN1 and SCD1. Phospho.ELM [78] database was used for the assessment of experimentally confirmed phosphorylation sites, as this database contains a collection of experimentally verified Ser, Thr and Tyr residues in eukaryotic proteins. In order to determine the surface accessibility of predicted phosphorylation sites, NetSurfP program [79] was used.

#### 4.9.2. Prediction of Ubiquitylation Sites in LC3A, LC3B, BECN1 and SCD1

Ubiquitylation (also known as Ubiquitination) is a modification process, in which a small regulatory protein ubiquitin is attached via an isopeptide bond to lysine residues of the substrate protein. Moreover, binding can occur either in the form of mono-ubiquitination (a single ubiquitin protein) or poly-ubiquitination (a chain of ubiquitin). Various regulatory roles of Ubiquitylation have been found, such as DNA repair, proteasomal degradation, transcription and signal transduction etc. we used three online web servers CKSAAP_UbSite (http://protein.cau.edu.cn/cksaap_ubsite/) [80], iUbiq-Lys (http://www.jci-bioinfo.cn/iUbiq-Lys) [81] and BDM-PUB (http://bdmpub.biocuckoo.org/prediction.php) [82] to predict Ubiquitylation sites in LC3A, LC3B, BECN1 and SCD1. Lysine residue predicted with high confidence in at least two softwares were considered to have high potential for ubiquitylation.

#### 4.9.3. Prediction of Palmitoylation and Methylation Sites

Online Java based server CSS-PALM 3.0 (http://csspalm.biocuckoo.org/index.php) [83] was used to find out potential palmitoylation sites with CXXC or CC pattern, where C represents a Cys residue and X represents a random residue. This web-server predicts probable sites on the basis of experimentally verified palmitoylation sites.

Protein methylation plays a key role in PPIs which are the central players in almost every cellular process. PMes program, a support vector machine and enhanced feature based encoding scheme (http://bioinfo.ncu.edu.cn/inquiries_PMeS.aspx) [84] was used for the prediction of potential methylation sites. PMes server has been reported to provide superior predictive robustness as compare to other existing methylation site predicting servers.

#### 4.9.4. Prediction of Acetylating Sites

Lysine acetylation has emerged as a major PTM for histones and in other nuclear proteins. It is considered as the best characterized PTM after phosphorylation [39]. It is a widespread reversible covalent modification in eukaryotes, transferring acetyl groups to lysines at specific sites. Specific histone modifications and cancer mutations have been previously reported to silence tumor suppressor genes and enhance cancer progression [85]. For the prediction of acetylation sites in LC3A, LC3B, BECN1 and SCD1, web servers ASEB (http://bioinfo.bjmu.edu.cn/huac/predict_p/) [86] and PAIL (http://bdmpail.biocuckoo.org/results.php) [87] were used.

#### 4.9.5. Prediction of Sumoylation Sites in LC3A, LC3B, BECN1 and SCD1

Sumoylation, an essential and reversible PTM has been known to be a contributing factor in various diseases and disorders. SUMOs are conjugated via formation of isopeptide bond with the amino group of a lysine side chain in the target protein. Lysine residues having the potential for SUMOylation are often present within the consensus sequence ΨKX(D/E), where Ψ represents hydrophobic amino acid and X represents any amino acid. Three prediction algorithms were used to identify potential SUMOylation sites in LC3A, LC3B, BECN1 and SCD1. Putative sumoylation sites were identified using the SUMOplot™ Analysis Program (http://www.abgent.com/sumoplot), SUMOsp 2.0 (http://sumosp.biocuckoo.org/) [88] and SUMOhydro (http://protein.cau.edu.cn/others/SUMOhydro/introduction.html) [89] servers. For SUMOplot, a threshold score of >0.6 was considered. Medium level threshold was chosen for SUMOsp 2.0 analysis.

### 4.10. PPI Analysis for LC3A, LC3B, BECN1 and SCD1

Recent findings have found that various disease related polymorphisms tend to propagate through the PPI network. Understanding how these variations can rewire the protein interaction network associated with intricate disorders, such as cancer is fundamental. The disruption of the PPI can be explained by a variety of structural consequences including the formation of a steric clash, the loss of salt bridges and hydrogen bonds, the destabilization due to diminution of the hydrophobic effect and alteration in the main-chain conformation [90]. Bioinformatic methods can play an important role in thorough examining of SNP-induced rewiring of a disease network. Many missense SNPs have been found in the vicinity of PPI interfaces ultimately disrupting the protein interaction complexes [91]. SNPs in LC3A, LC3B, BECN1 and SCD1 affecting the PPI study was performed by PinSnps, a tool to generate comprehensive mapping of available SNPs onto PPI (see Figure S1).

## Figures and Tables

**Table 1:** Missense SNPs in LC3A, LC3B, BECN1 and SCD1 predicted to be deleterious using nsSNP Analyzer, PROVEAN, PMUT and SNPs & GO. I-Mutant2.0, DUET and STRUM web servers were used for stability analysis.

Protein	Mutation	Deleterious SNPs				Destabilizing SNPs		
		nsSNP Analyzer	PROVEAN	PMUT	SNPs & GO.	I-Mutant2.0	DUET	STRUM
**LC3A**	R24C	√	√	√	√	√	√	√
	S29R	√	–	√	√	√	√	√
	P55A	√	√		√	√	√	√
	P55L	√	√	√	√	√	√	√
	R70C	√	√	√	√	√	√	√
	P75R	–	√	√	√	√	–	–
	F79V	√	√	√	√	√	√	√
	F79S	√	√	√	√	√	√	√
	N84K	–	√	√	√	√	–	√
	A96P	–	√	√	√	√	√	√
	D104N	√	√	–	√	√	√	√
	E105K	–	√	√	√	√	–	√
	K49A	√	√	√	√	√	√	√
	K51A	√	√	√	√	√	√	√
	L53A	√	√		√	√	√	√
	G120A	√	√	√	√	√	√	√
**LC3B**	R11C	√	√	√	√	√	√	√
	R24Q		√	√	√	√	√	√
	P32L	√	√	√	√	√	√	√
	V33A	√	√		√	√	√	√
	R37Q	√	√	√	√	√	√	√
	G40C	√	√	√	√	√	√	√
	R68A	√	√	√	√	√	√	√
	R70A	√	√	√	√	√	√	√
	R70H	√	√	√	√	√	√	√
	A78D	–	√	√	√	√	√	√
	F79S	√	√	√	√	√	√	√
	S92C	–	√	√	√	√	√	√
	D104N	–	√	√	√	√	√	√
	D106G	√	√	√	√	√	√	√
	Y113S	√	√	√	√	√	√	√
	Y113C	√	√	√	√	–	√	√
	E117V	–	√	√	√	–	√	√
	G120A	√	√	√	√	–	√	√
**BECN1**	V14E	√	√	–	√	√	√	√
	A44P	–	√	√	√		√	–
	R74C	√	–	√	√	√	√	√
	L112R	√	√	√	√	√	–	√
	S113R	√	√	√	√	–	√	√
	S127L	√	√	–	√	√	–	√
	L144F	√	√	√	–	√	√	√
	E156V	–	√	√	√	√	–	√
	R164C	√	√	√	√	√	√	√
	L194P	√	√	√	√	√	√	√
	L198P	–	√	√	√	√	√	√
	R205C	–	√	√	√	√	√	√
	Q239P	–	√	√	√	√	–	√
	R255C	√	√	√	√	√	√	√
	N268S	√	√	–	√	√	–	√
	T284S	√	√	–	√	√	√	√
	R292C	√	√	√	√	√	√	√
	R292H	√	√	√	√	√	√	√
	V298M	√	√	–	√	√	√	√
	V298A	√	√	–	√	√	√	√
	E302K	√	√	√	√	√	√	√
	L314H	√	√	√	√	√	√	√
	L323P	–	√	√	√	√	√	√
	Y338C	√	√	√	√	√	√	√
	S346Y	√	√	√	–	–	√	√
	P350L	√	√	√	–	√	–	√
	Y352H	√	√	–	√	√	√	√
	C353Y	√	√	√	√	√	√	√
	W361R	–	√	√	√	√	√	√
	C375R	√	√	√	√	√	√	√
	I403S	√	√	√	√	√	√	√
	I403T	√	√	√	√	√	√	√
	G408R	√	√	√	–	√	√	√
	W425C	√	√	√	√	√	√	√
	F431V	√	√	√	√	√	√	√
	F123A	√	√	√	√	√	√	√
	D133A	√	√	√	√	√	–	√
	D149A	–	√	√	√	√	–	√
	Y352A	√	√	√	√	√	√	√
	W425A	√	√	√	√	√	√	√
	Y41C	√	–	√	√	√	√	√
**SCD1**	P48R	√	–	√	√	√	√	√
	Y55D	√	–	√	√	√	√	√
	E63G	√	–	√	√	√	√	√
	Y88C	√	√	√	√	√	√	√
	G89R	√	√	√	√	√	√	–
	G89A	√	√	–	√	√	√	–
	T100I	√	√	√	√	√	–	–
	L102P	√	√	–	√	√	√	√
	G104E	√	–	√	√	√	√	√
	R121C	√	√	√	√	√	√	√
	H125P	√	√	√	√	–	√	–
	R126C	√	√	–	√	√	√	√
	R126S	√	√	√	√	√	√	√
	R131W	√	√	√	√	√	√	√
	R135W	√	√	√	√	√	√	√
	R135Q	–	√	√	√	√	√	√
	M144T	√	√	√	√	√	√	√
	Y151C	√	√	√	√	–	√	√
	R188C	√	√	–	√	√	√	√
	H190Y	√	√	–	√	–	–	√
	F213L	√	√	–	√	√	√	√
	Q214R	√	√	–	√	√	√	√
	Y218H	√	√	–	√	√	√	√
	Y218C	√	√	√	√	√	√	√
	M225T	√	√	–	√	√	√	√
	T231M	√	√	–	√	√	√	√
	W238G	√	√	√	√	√	√	√
	W238R	√	√	√	√	√	√	√
	A249D	–	√	√	√	√	√	√
	G272R	√	√	√	√	√	√	–
	Y273C	√	–	√	√	√	√	√
	N279I	–	√	√	√	–	–	√
	R283W	√	√	√	√	√	√	√
	L290P	√	√	–	√	√	√	√
	F323V	√	√	√	√	√	√	√
	C326G	√	√	√	√	√	√	√
	C326F	√	–	√	√	√	√	√
	M327V	–	√	√	√	√	√	√
	G331S	√	√	√	√	√	√	√
	A333T	–	√	√	√	√	√	–
	R336Q	√	–	√	√	√	√	√
	R336L	√	–	√	√	√	–	–

Missense SNPs with 3 or more deleterious predictions are considered high-risk SNPs; SNPs were considered as destabilizing in nature if two or more than two algorithms showed a decrease in stability upon mutation. √, Yes; –, No.

## Supplementary Materials

Supplementary materials can be found at www.mdpi.com/1422-0067/18/1/139/s1.
