# Supplementary material for: Mutation-Structure-Function Relationship Based Integrated Strategy Reveals the Potential Impact of Deleterious Missense Mutations in Autophagy Related Proteins on Hepatocellular Carcinoma (HCC): A Comprehensive Informatics Approach
Source: Int J Mol Sci. 2017 Jan 11;18(1):139. doi: 10.3390/ijms18010139 (PMC5297772; doi:10.3390/ijms18010139)
Supplement: Supplementary file 1 [file ijms-18-00139-s001.pdf]

Supplementary Materials: Mutation-Structure-Function Relationship Based Integrated Strategy Reveals the Potential Impact of Deleterious Missense Mutations in Autophagy Related Proteins on Hepatocellular Carcinoma (HCC): A Comprehensive Informatics Approach

Faryal Mehwish Awan, Ayesha Obaid, Aqsa Ikram and Hussnain Ahmed Janjua

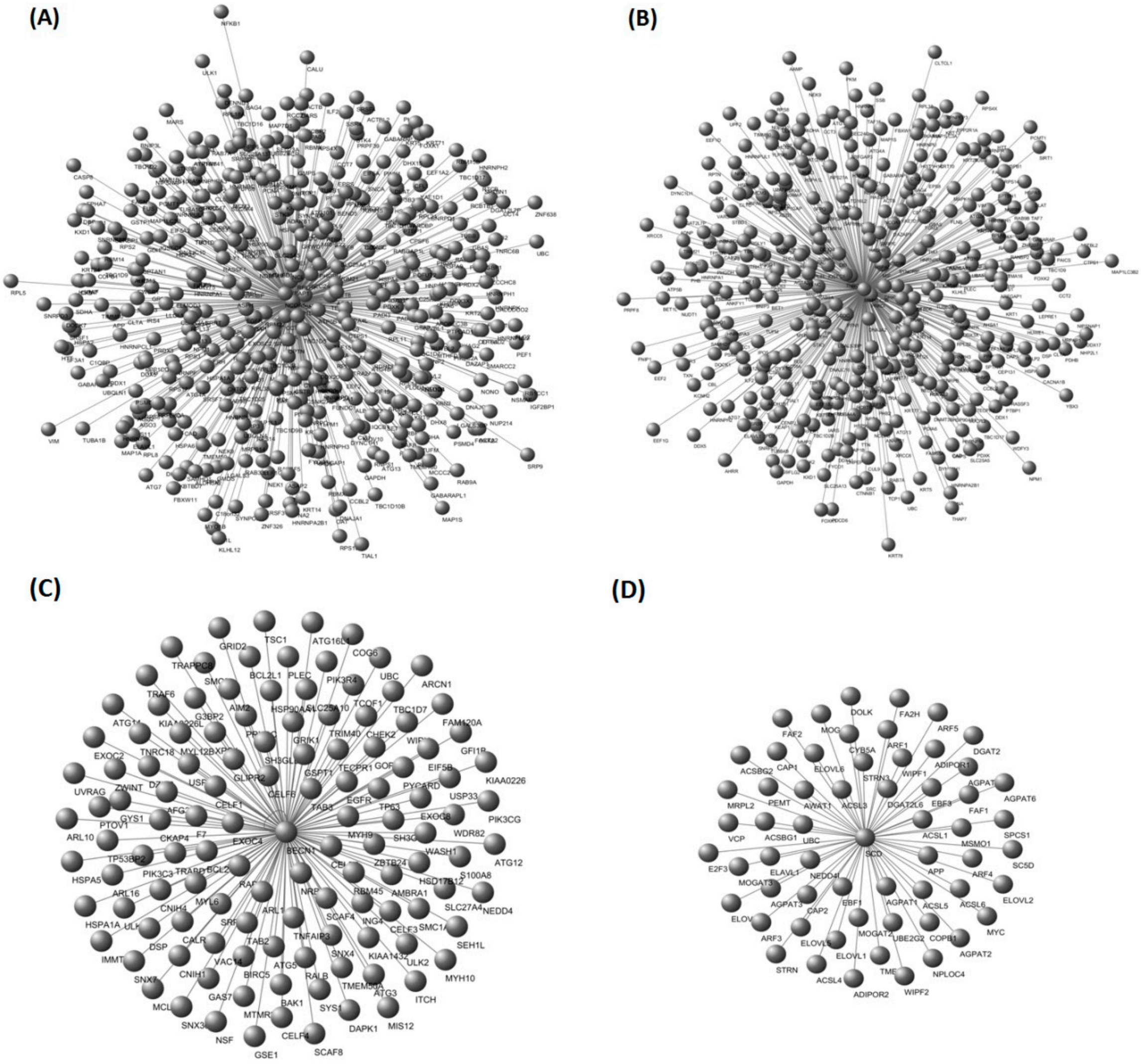

Figure S1. Visualization of the protein-protein interaction sub network of LC3A (**A**), LC3B (**B**), BECN1 (**C**) and SCD1 (**D**) proteins using PinSnps tool. Illustration created using Cytoscape, an open source platform for visualizing molecular interaction networks. The connection between two nodes is indicated by an edge.

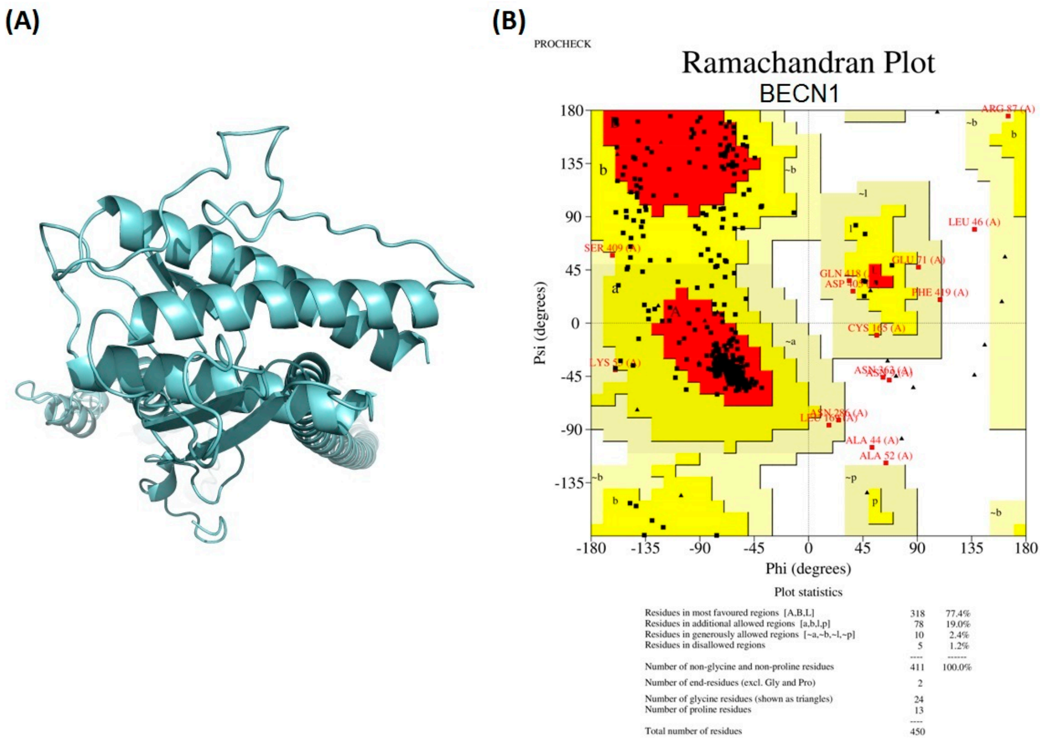

Figure S2. (**A**) Cartoon representation of the top modeled 3D structure of BECN1 built from I-TASSER server; (**B**) The Ramachandran plot calculation of the psi/phi angle distribution of BECN1 model using PROCHECK validation server showing the stereochemical quality of the structure. The most favored regions are marked as (A, B, L). The additional allowed regions are marked as {a, b, l, p}. All non-glycine and proline residues are shown as filled black squares, whereas, glycines (non-end) are shown as filled black triangles. Disallowed residues are colored red.

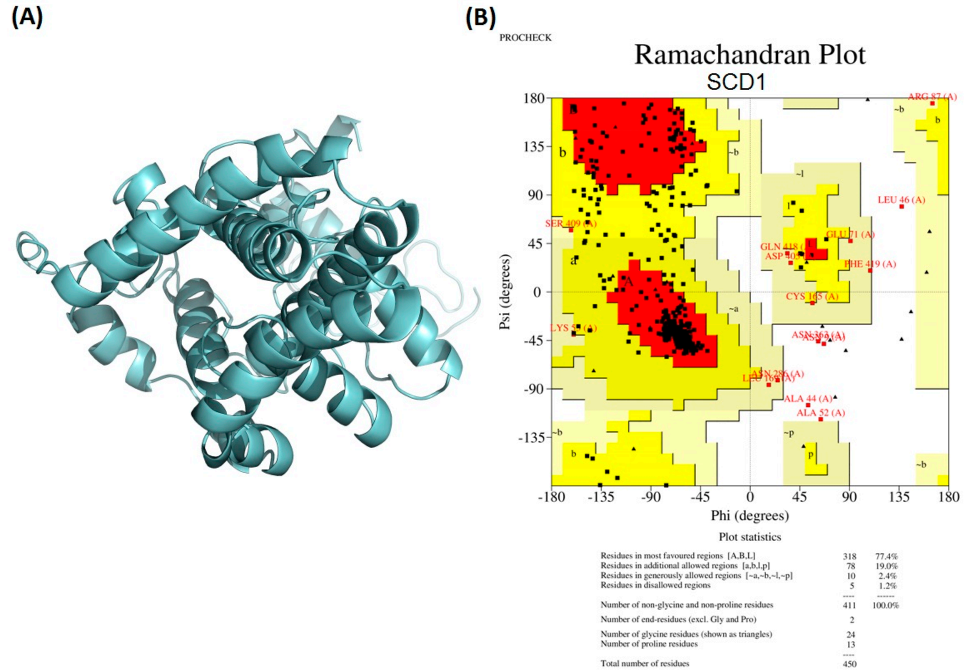

Figure S3. (**A**) Cartoon representation of the top modeled 3D structure of SCD1 built from I-TASSER server; (**B**) The Ramachandran plot calculation of the psi/phi angle distribution of SCD1 model using PROCHECK validation server showing the stereochemical quality of the structure. The most favored regions are marked as (A, B, L). The additional allowed regions are marked as {a, b, l, p}. All non-glycine and proline residues are shown as filled black squares, whereas, glycines (non-end) are shown as filled black triangles. Disallowed residues are colored red.

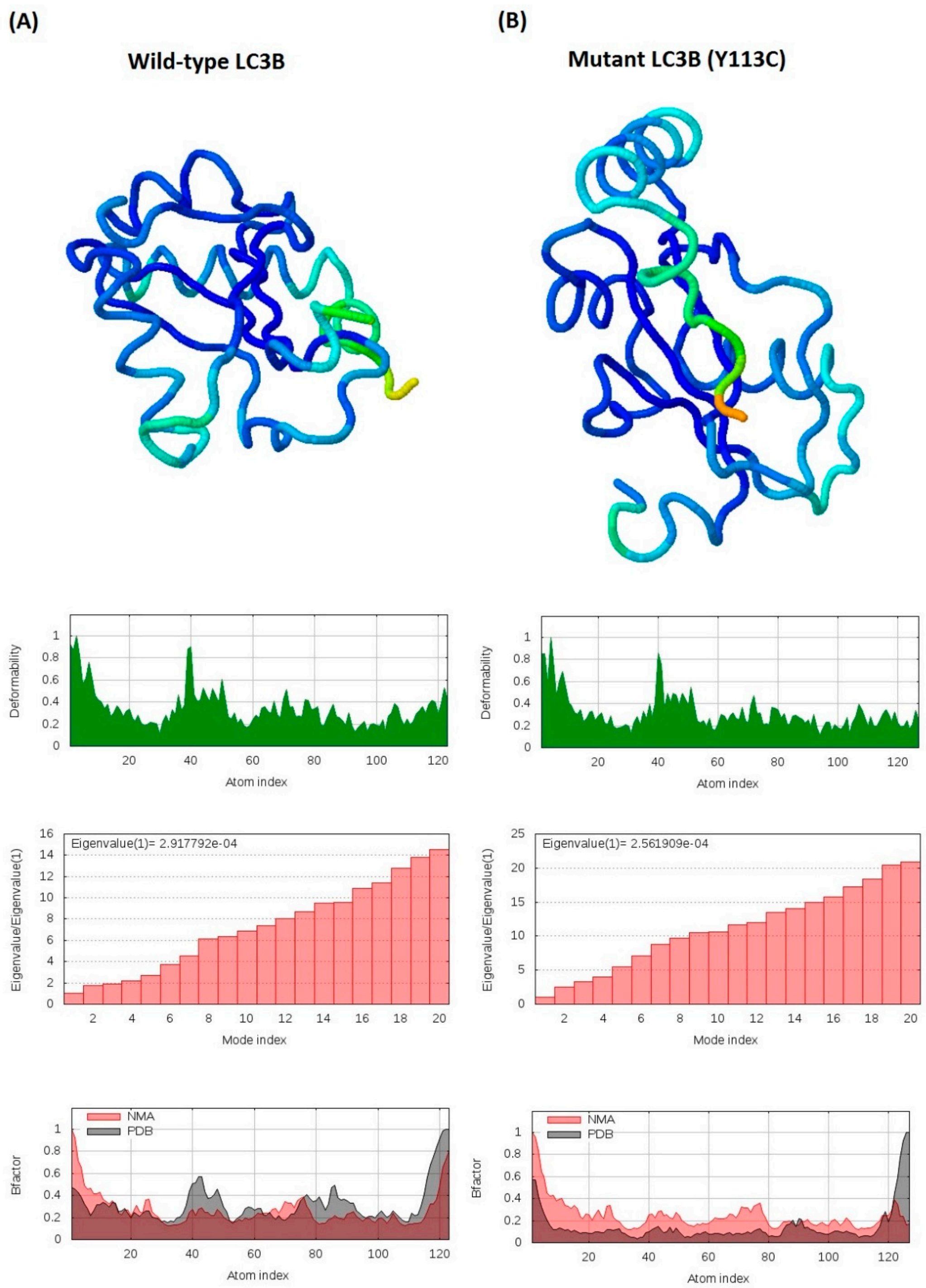

Figure S4. Normal mode analysis of WT (**A**) and mutant LC3B (Y113C) (**B**) protein. Detailed profiles of mobility (B-factors), eigenvalues and deformability have been shown.

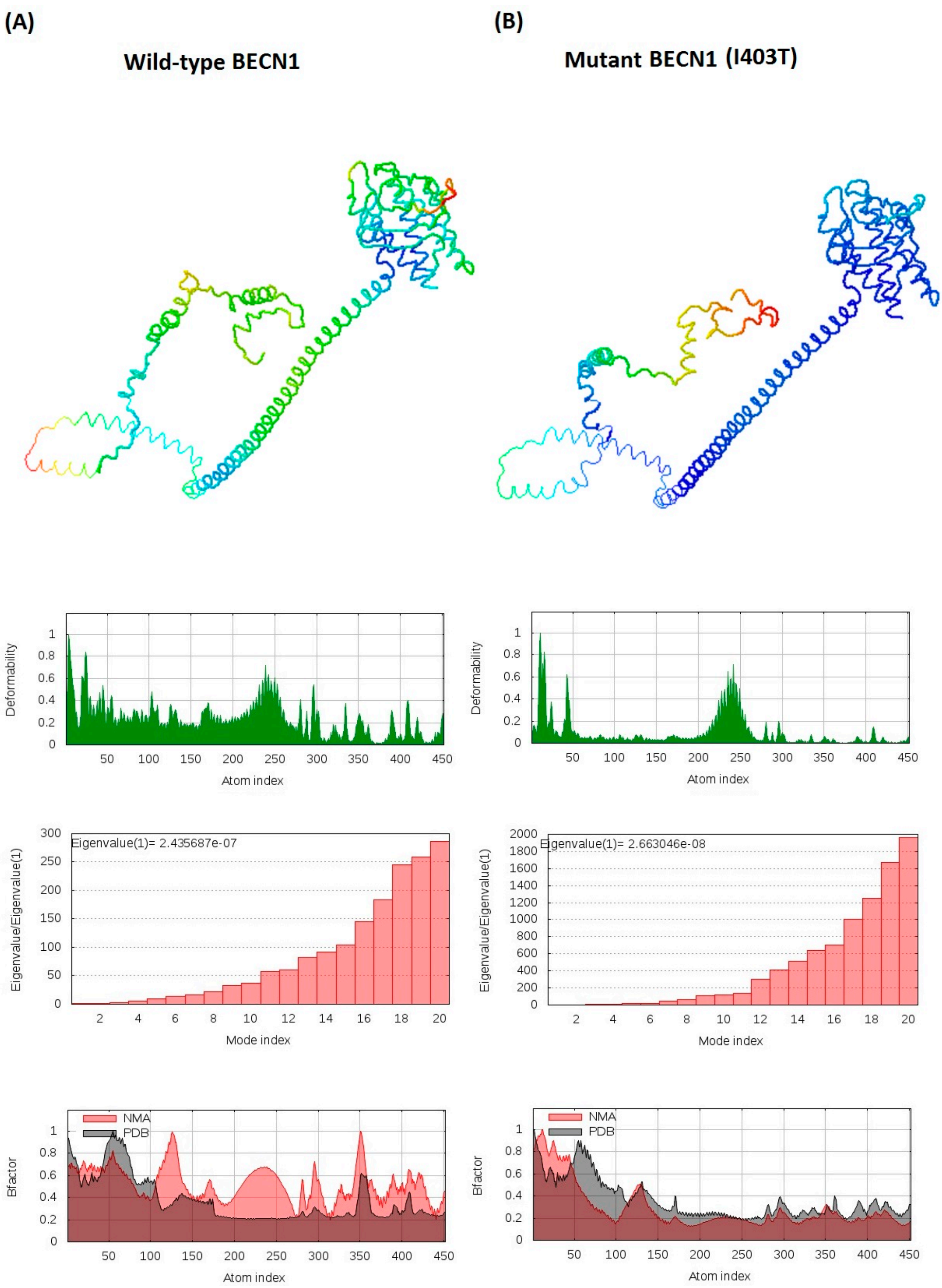

Figure S5. Normal mode analysis of WT (**A**) and mutant BECN1 (I403T) (**B**) protein. Detailed profiles of mobility (B-factors), eigenvalues and deformability have been shown.

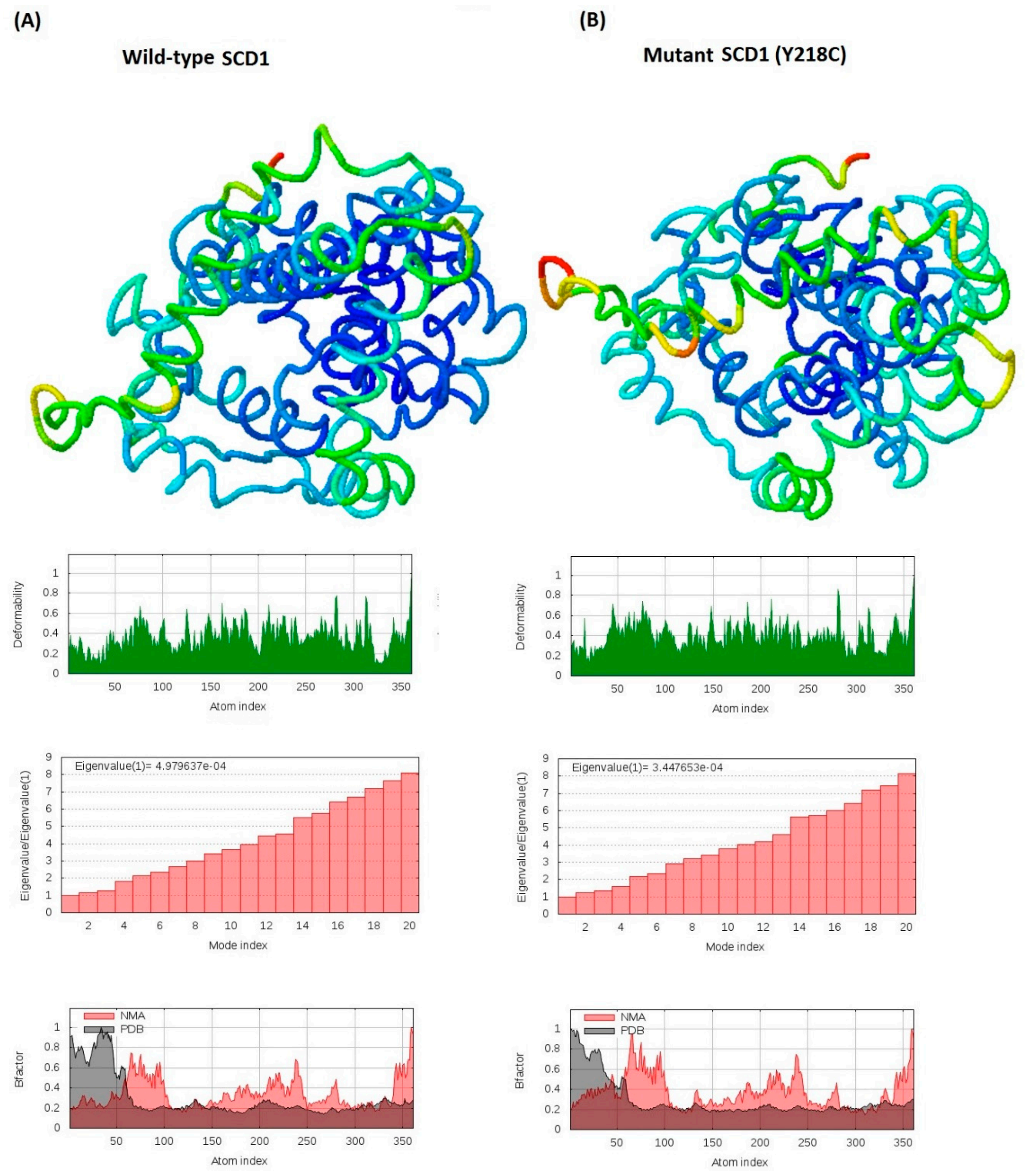

Figure S6. Normal mode analysis of WT (**A**) and mutant SCD1 (Y218C) (**B**) protein. Detailed profiles of mobility (B-factors), eigenvalues and deformability have been shown.

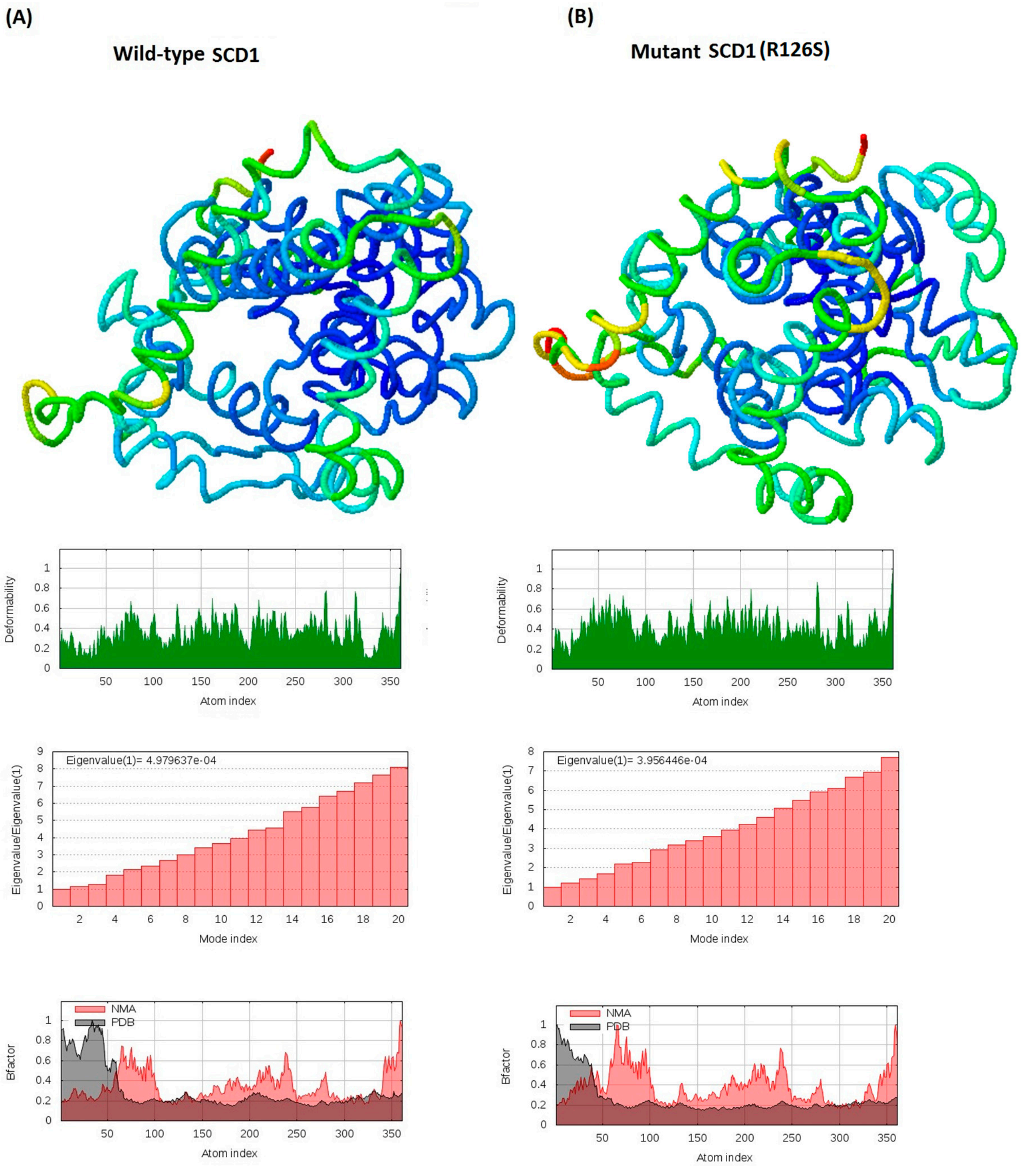

Figure S7. Normal mode analysis of WT (**A**) and mutant SCD1 (R126S) (**B**) protein. Detailed profiles of mobility (B-factors), eigenvalues and deformability have been shown.

Table S1. Evaluating the effect of the amino acid mutations in LC3A by HOPE sever. The distinct pattern and possible consequences of native and mutant amino acid on protein structure based on size, charge, and hydrophobicity have been shown.

WT Amino Acid	Position	M amino Acid	Characteristics and Possible Consequences
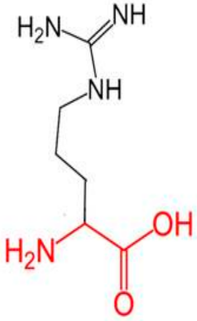 **Arg**	24	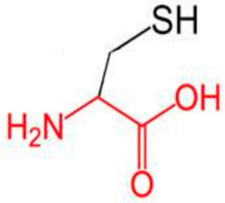 Cys	The mutant (M) residue is smaller and more hydrophobic than the wild-type (WT) residue. The WT residue was +vely charged, the M residue is neutral. The WT residue forms a salt bridge with Asp at position 25. The difference in charge will disturb the ionic interactions.
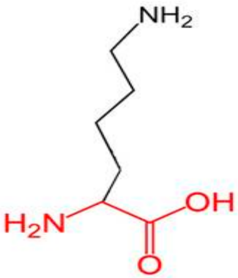 **Lys**	49	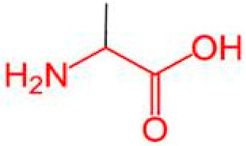 Ala	The M residue is smaller and more hydrophobic than the WT residue. The WT residue was +vely charged, the M residue is neutral. The WT residue forms a salt bridge with: Asp at position 48. The difference in charge will disturb the ionic interactions.
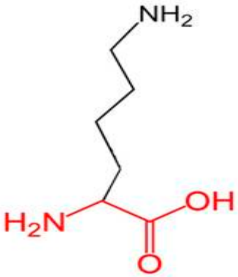 **Lys**	51	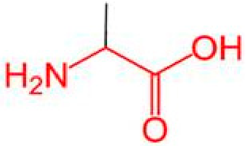 Ala	The WT residue forms a hydrogen bond with Asp at position 4 and Thr at position 50. The size difference between WT and M residue makes that the new residue is not in the correct position to make the same hydrogen bond as the original WT residue did. The difference in hydrophobicity will affect hydrogen bond formation. The WT residue forms a salt bridge with Asp at position 4, Glu at position 19 and Glu at position 36. The difference in charge will disturb the ionic interactions.

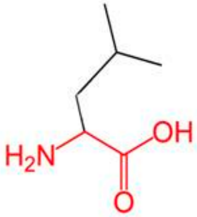 **Leu**	53	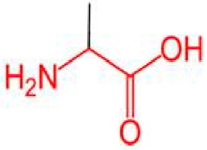 Ala	The M residue is smaller than the WT residue. This will cause a probable loss of external interactions.
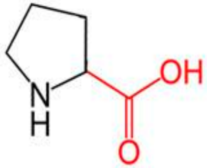 **Pro**	55	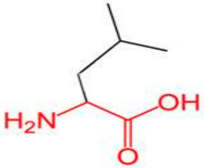 Leu	The M residue is bigger than the WT residue. The WT residue is a Pro. Pro is known to be very rigid and therefore induce a special backbone conformation which might be required at this position. The mutation can disturb this special conformation. The residue is located on the surface of the protein; mutation of this residue can disturb interactions with other molecules or other parts of the protein.
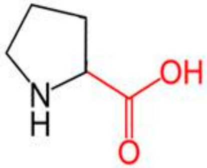 **Pro**	55	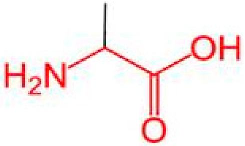 Ala	The M residue is smaller than the WT residue. The WT residue is a Pro. Pros are known to be very rigid and therefore induce a special backbone conformation which might be required at this position. The mutation can disturb this special conformation.
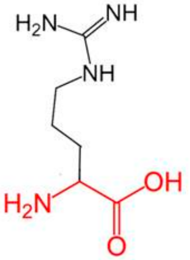 **Arg**	70	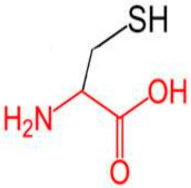 Cys	The M residue is smaller and more hydrophobic than the WT residue. The WT residue was +vely charged, the M residue is neutral. The WT residue forms a salt bridge with: Asp at position 48. The difference in charge will disturb the ionic interactions.

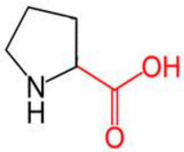 **Pro**	75	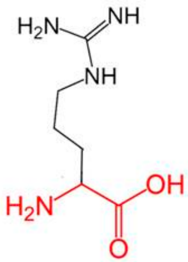 Arg	The WT residue was neutral, the M residue is +vely charged. The mutation introduces a charge at this position; this can cause repulsion between the M residue and neighboring residues. The residue is located on the surface of the protein; mutation of this residue can disturb interactions with other molecules or other parts of the protein.
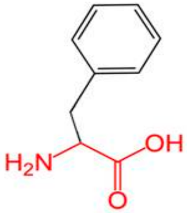 **Phe**	79	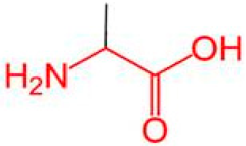 Ala	The residue is buried in the core of a domain. The differences between the WT and M residue might disturb the core structure of this domain. The mutation will cause an empty space in the core of the protein.
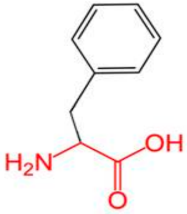 **Phe**	79	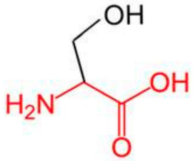 Ser	The mutation will cause an empty space and loss of hydrophobic interactions in the core of the protein. The WT residue is predicted to be located in its preferred secondary structure, a β-strand. The M residue prefers to be in another secondary structure; therefore the local conformation will be slightly destabilized.
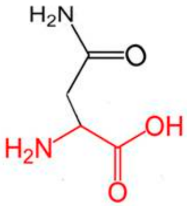 **Asn**	84	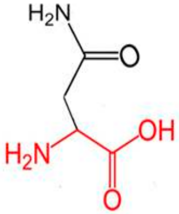 Lys	The WT residue forms a hydrogen bond with Glu at position 102. The size difference between WT and M residue makes that the new residue is not in the correct position to make the same hydrogen bond as the original WT residue did. The WT residue is involved in a multimer contact. The mutation introduces a bigger residue at this position; this can disturb the multimeric interactions. M residue will cause slight destabilization in local conformation. The mutation introduces a charge at this position; this can cause repulsion between the M residue and neighboring residues. The residue is located on the surface of the protein; mutation of this residue can disturb interactions with other molecules or other parts of the protein.

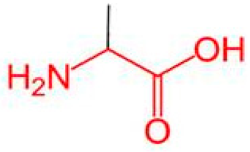<br>**Ala**	96	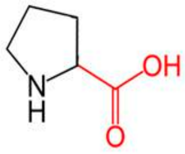<br>Pro	The WT residue is predicted (by the PHDsec DAS-server) to be located in an α-helix.<br>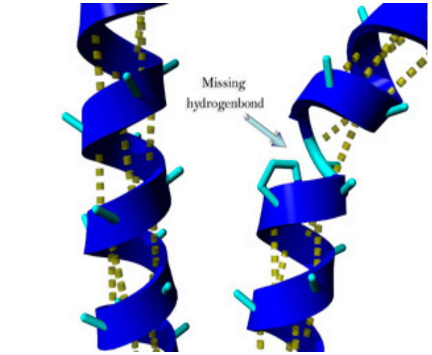<br><br>Pro disrupts an α-helix when not located at one of the first 3 positions of that helix. In case of the mutation at hand, the helix will be disturbed and this can have severe effects on the structure of the protein.
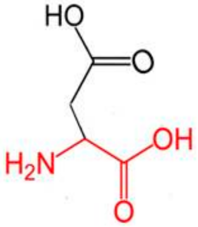<br>**Asp**	104	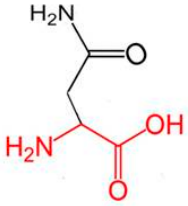<br>Asn	The WT residue forms a hydrogen bond with Asp at position 106, and a salt bridge with Lys at position 8 and Arg at position 16. The difference in charge will disturb the ionic interaction made by the original, WT residue.
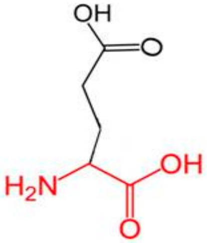<br>**Glu**	105	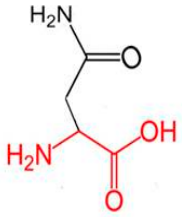<br>Lys	The WT residue forms a salt bridge with Arg at position 16. The difference in charge will disturb the ionic interaction made by the original, WT residue. The residue is located on the surface of the protein; mutation of this residue can disturb interactions with other molecules or other parts of the protein.

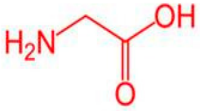 **Gly**	120	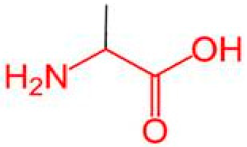 Ala	The WT residue is a Gly, the most flexible of all residues. This flexibility might be necessary for the protein's function. Mutation of this Gly can abolish this function. Residues in the vicinity of the mutated residue are annotated in the UniProt database as being a binding site. The mutation could affect the local structure and as a consequence affect this binding site.

Table S2. Evaluating the effect of the amino acid mutations in LC3B by HOPE sever. The distinct pattern and possible consequences of native and Mutant amino acid on protein structure based on size, charge, and hydrophobicity have been shown.

WT Amino Acid	Position	M amino Acid	Characteristics and Possible Consequences
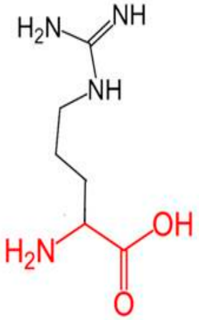 **Arg**	11	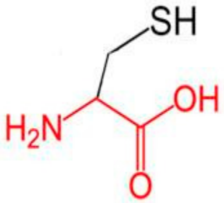 Cys	The M residue is smaller and more hydrophobic than the WT residue. The WT residue was +vely charged, the M residue is neutral. The WT residue forms a hydrogen bond and salt bridge with Asp at position 19. The difference in hydrophobicity will affect hydrogen bond formation. The difference in charge will disturb the ionic interaction made by the original, WT residue.
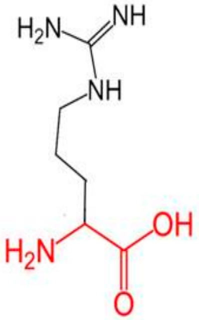 **Arg**	24	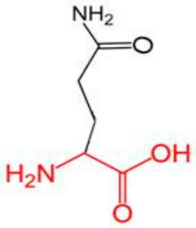 Gln	The M residue is smaller than the WT residue. The WT residue was +vely charged, the M residue is neutral. The WT residue forms a salt bridge with Glu at position 100. The difference in charge will disturb the ionic interaction made by the original, WT residue.

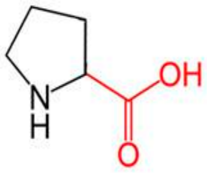 **Pro**	32	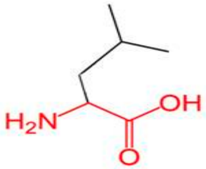 Leu	The M residue is bigger than the WT residue. The WT residue is a Pro. Pro is known to be very rigid and therefore induce a special backbone conformation which might be required at this position. The mutation can disturb this special conformation. The residue is buried in the core of a domain. The differences between the WT and M residue might disturb the core structure of this domain.
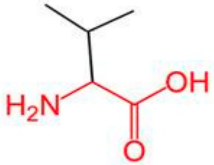 **Val**	33	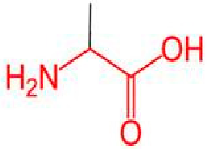 Ala	The M residue is smaller than the WT residue. The mutation will cause an empty space in the core of the protein.
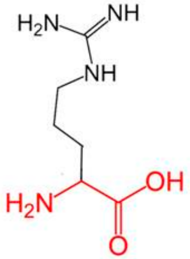 **Arg**	37	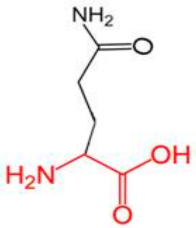 Gln	The M residue is smaller and more hydrophobic than the WT residue. The WT residue was +vely charged, the M residue is neutral. The WT residue forms a hydrogen bond with Leu at position 44 and a salt bridge with Glu at position 41. The difference in charge will disturb the ionic interaction made by the original, WT residue.
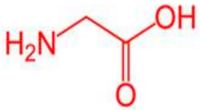 **Gly**	40	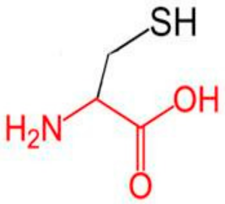 Cys	The M residue is bigger and more hydrophobic than the WT residue. The WT residue is a Pro. The WT residue is a Gly, the most flexible of all residues. This flexibility might be necessary for the protein's function. Mutation of this Gly can abolish this function. The residue is located on the surface of the protein; mutation of this residue can disturb interactions with other molecules or other parts of the protein. The torsion angles for this residue are unusual. Only Gly is flexible enough to make these torsion angles, mutation into another residue will force the local backbone into an incorrect conformation and will disturb the local structure.

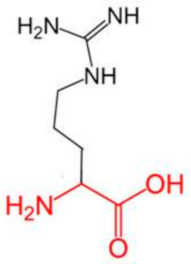 **Arg**	68	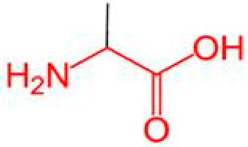 Ala	The M residue is smaller and more hydrophobic than the WT residue. The WT residue was +vely charged, the M residue is neutral. The charge of the WT residue is lost by this mutation. This can cause loss of interactions with other molecules.
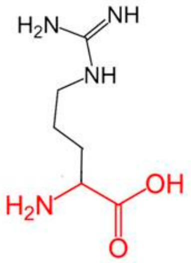 **Arg**	70	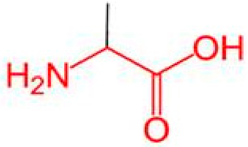 Ala	The M residue is smaller and more hydrophobic than the WT residue. The WT residue was +vely charged, the M residue is neutral. The charge of the WT residue is lost by this mutation. This can cause loss of interactions with other molecules. Any hydrogen bond that could be made by the WT residue to other monomers will be lost now and affect the multimeric contacts.
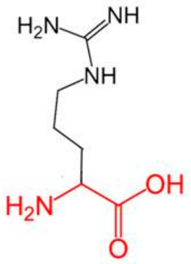 **Arg**	70	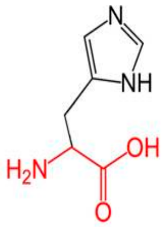 His	The M residue is smaller than the WT residue. The WT residue was +vely charged, the M residue is neutral. The charge of the WT residue is lost by this mutation. This can cause loss of interactions with other molecules. Any hydrogen bond that could be made by the WT residue to other monomers will be lost now and affect the multimeric contacts.
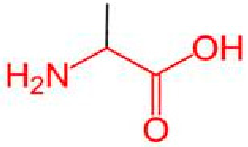 **Ala**	78	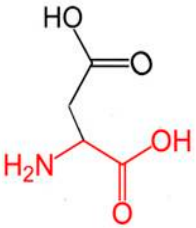 Asp	The M residue is bigger and less hydrophobic than the WT residue. The WT residue was neutral, the M residue is -vely charged. The mutation introduces a charge at this position; this can cause repulsion between the M residue and neighboring residues.

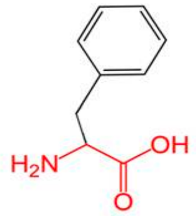 **Phe**	79	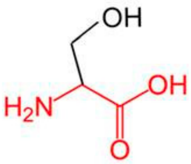 Ser	The M residue is smaller and more hydrophobic than the WT residue. The WT residue was +vely charged, the M residue is neutral. The charge of the WT residue is lost by this mutation. The residue is buried in the core of a domain. The differences between the WT and M residue might disturb the core structure of this domain.
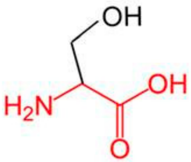 **Ser**	92	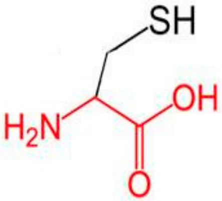 Cys	The M residue is more hydrophobic than the WT residue. The WT residue forms a hydrogen bond with: Ser at position 90. The difference in hydrophobicity will affect hydrogen bond formation.
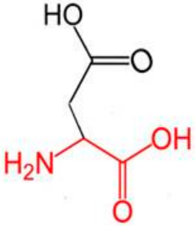 **Asp**	104	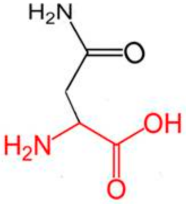 Asn	The WT residue was -vely charged, the M residue is neutral. The WT residue forms a hydrogen bond with Lys at position 8 and Asp at position 106. The WT residue forms a salt bridge with Lys at position 8 and Arg at position 16. The difference in charge will disturb the ionic interaction made by the original, WT residue.
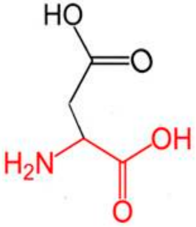 **Asp**	106	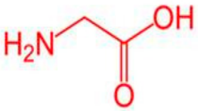 Gly	The M residue is smaller and more hydrophobic than the WT residue. The WT residue was -vely charged, the M residue is neutral. The mutation introduces a Gly at this position. Glys are very flexible and can disturb the required rigidity of the protein at this position. The WT residue forms a hydrogen bond with Arg at position 16, Phe at position 108 and Tyr at position 110. The difference in hydrophobicity will affect hydrogen bond formation. The WT residue forms a salt bridge with Lys at position 8 and Arg at position 16.

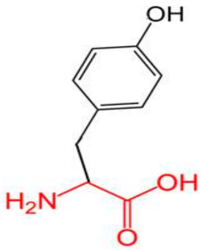 **Tyr**	113	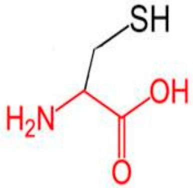 Cys	The M residue is smaller than the WT residue. The M residue is more hydrophobic than the WT residue. This will cause a possible loss of external interactions. The mutation will cause loss of hydrogen bonds in the core of the protein and as a result disturb correct folding.
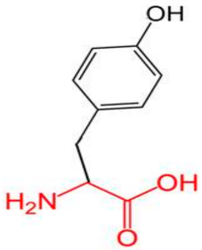 **Tyr**	113	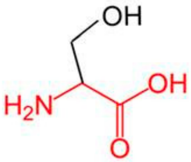 Ser	The M residue is smaller than the WT residue. The mutation will cause an empty space in the core of the protein.
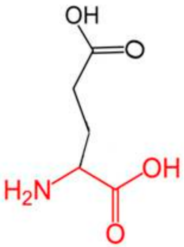 **Glu**	117	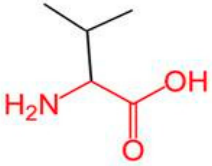 Val	The M residue is smaller and more hydrophobic than the WT residue. The WT residue was -vely charged, the M residue is neutral. The WT residue forms a salt bridge with Lys at position 42. The difference in charge will disturb the ionic interaction made by the original, WT residue. The mutation converts the WT residue in a residue that does not prefer α-helices as secondary structure.
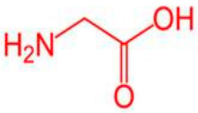 **Gly**	120	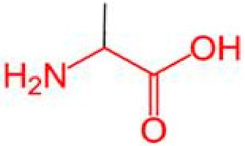 Ala	The WT residue is a Gly, the most flexible of all residues. This flexibility might be necessary for the protein's function. Mutation of this Gly can abolish this function. The WT and M amino acids differ in size. The M residue is bigger than the WT residue. The residue is located on the surface of the protein; mutation of this residue can disturb interactions with other molecules or other parts of the protein. The torsion angles for this residue are unusual. Only Gly is flexible enough to make these torsion angles, mutation into another residue will force the local backbone into an incorrect conformation and will disturb the local structure.

Table S3. Evaluating the effect of the amino acid mutations in BECN1 by HOPE sever. The distinct pattern and possible consequences of native and Mutant amino acid on protein structure based on size, charge, and hydrophobicity have been shown.

WT Amino Acid	Position	M Amino Acid	Characteristics and Possible Consequences
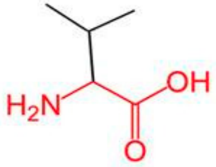 **Val**	14	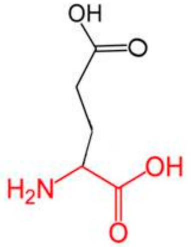 Glu	The M residue is bigger and less hydrophobic than the WT residue. The WT residue is predicted (by the PHDsec DAS-server) to be located in its preferred secondary structure, a β-strand. The M residue prefers to be in another secondary structure; therefore the local conformation will be slightly destabilized. The mutation introduces a charge; this can cause repulsion of ligands or other residues with the same charge.
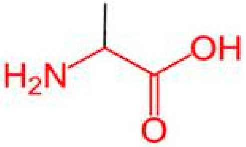 **Ala**	44	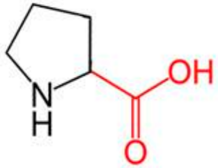 Pro	The M residue is bigger than the WT residue. This might lead to bumps.
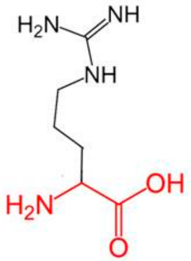 **Arg**	74	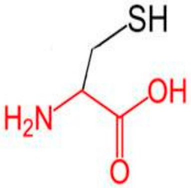 Cys	The mutation introduces a more hydrophobic residue at this position. This can result in loss of hydrogen bonds and/or disturb correct folding. The charge of the WT residue will be lost, this can cause loss of interactions with other molecules or residues.
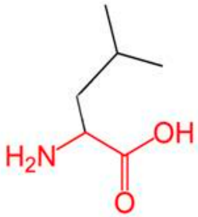 **Leu**	112	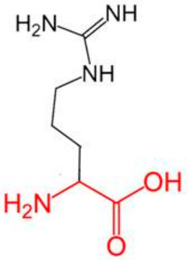 Arg	In both the PDB-file and in the PISA-assembly, this residue was found to be involved in a multimer contact. The mutation introduces a bigger residue at this position, this can disturb the multimeric interactions. The mutation introduces a less hydrophobic residue. Sometimes, hydrophobicity is important for multimerisation and therefore this mutation could affect the multimer contacts. The residue is located on the surface of the protein; mutation of this residue can disturb interactions with other molecules or other parts of the protein. The mutation introduces a charge at this position, this can cause repulsion between the M residue and neighboring residues.

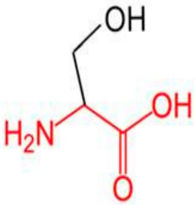 **Ser**	113	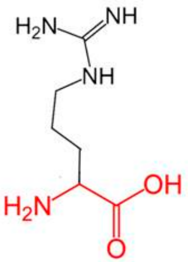 Arg	The WT residue forms a hydrogen bond with: Val at position 14, Val at position 14. The size difference between WT and M residue makes that the new residue is not in the correct position to make the same hydrogen bond as the original WT residue did. The difference in hydrophobicity will affect hydrogen bond formation. In both the PDB-file and in the PISA-assembly, this residue was found to be involved in a multimer contact. The mutation introduces a bigger residue at this position, this can disturb the multimeric interactions. The mutation introduces a less hydrophobic residue. Sometimes, hydrophobicity is important for multimerisation and therefore this mutation could affect the multimer contacts.
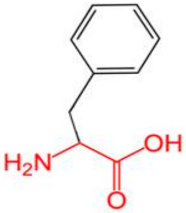 **Phe**	123	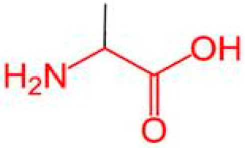 Ala	In both the PDB-file and in the PISA-assembly, this residue was found to be involved in a multimer contact. The mutation introduces a smaller residue at this position. The new residue might be too small to make multimer contacts.
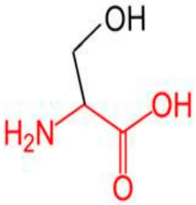 **Ser**	127	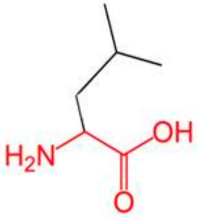 Leu	The WT residue forms a hydrogen bond with: Asp at position 124. The size difference between WT and M residue makes that the new residue is not in the correct position to make the same hydrogen bond as the original WT residue did. The difference in hydrophobicity will affect hydrogen bond formation. In both the PDB-file and in the PISA-assembly, this residue was found to be involved in a multimer contact. The mutation introduces a bigger residue at this position; this can disturb the multimeric interactions. A more hydrophobic residue is introduced here. Any hydrogen bond that could be made by the WT residue to other monomers will be lost now and affect the multimeric contacts.
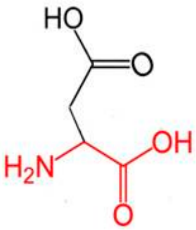 **Asp**	133	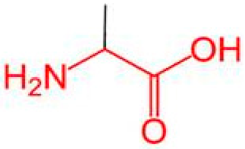 Ala	The charge of the WT residue will be lost; this can cause loss of interactions with other molecules or residues. The mutation introduces a more hydrophobic residue at this position. This can result in loss of hydrogen bonds and/or disturb correct folding.

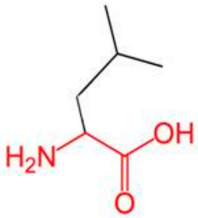 **Leu**	144	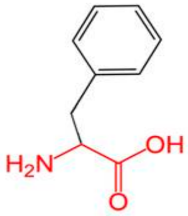 Phe	The WT residue is predicted (by the PHDsec DAS-server) to be located in an α-helix. The mutation converts the WT residue in a residue that does not prefer α-helices as secondary structure. The residue is located on the surface of the protein, mutation of this residue can disturb interactions with other molecules or other parts of the protein.
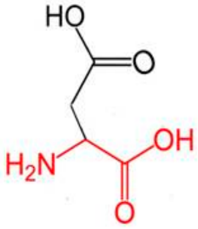 **Asp**	149	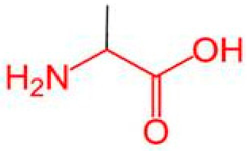 Ala	In the PDB file used for this analysis the mutated residue is involved in a multimer contact. The mutation introduces a smaller residue at this position. The new residue might be too small to make multimer contacts. A more hydrophobic residue is introduced here. Any hydrogen bond that could be made by the WT residue to other monomers will be lost now and affect the multimeric contacts.
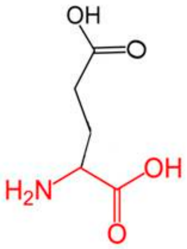 **Glu**	156	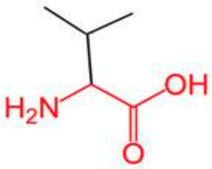 Val	In the PDB file used for this analysis the mutated residue is involved in a multimer contact. The mutation introduces a smaller residue at this position. The new residue might be too small to make multimer contacts. A more hydrophobic residue is introduced here. Any hydrogen bond that could be made by the WT residue to other monomers will be lost now and affect the multimeric contacts.
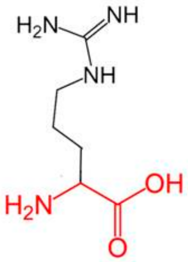 **Arg**	164	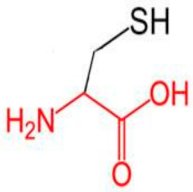 Cys	The WT residue forms a salt bridge with: Glu at position 167. The difference in charge will disturb the ionic interaction made by the original, WT residue.

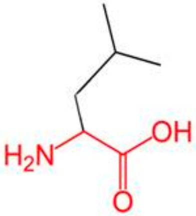 **Leu**	194	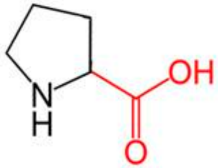 Pro	The WT residue is predicted (by the PHDsec DAS-server) to be located in an α-helix. Pro disrupts an α-helix when not located at one of the first 3 positions of that helix. In case of the mutation at hand, the helix will be disturbed and this can have severe effects on the structure of the protein.
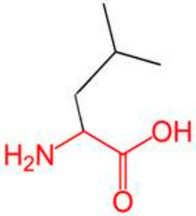 **Leu**	198	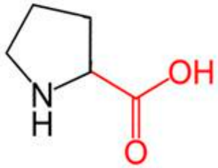 Pro	The WT residue is predicted (by the PHDsec DAS-server) to be located in an α-helix. Pro disrupts an α-helix when not located at one of the first 3 positions of that helix. In case of the mutation at hand, the helix will be disturbed and this can have severe effects on the structure of the protein.
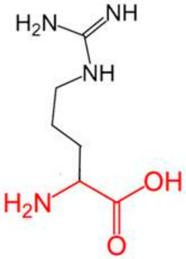 **Arg**	205	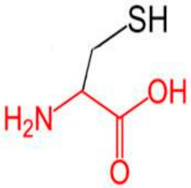 Cys	The WT residue forms a salt bridge with: Glu at position 202. The difference in charge will disturb the ionic interaction made by the original, WT residue. In both the PDB-file and in the PISA-assembly, this residue was found to be involved in a multimer contact. The mutation introduces a smaller residue at this position. The new residue might be too small to make multimer contacts. A more hydrophobic residue is introduced here. Any hydrogen bond that could be made by the WT residue to other monomers will be lost now and affect the multimeric contacts.

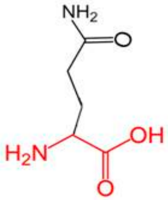 **Gln**	239	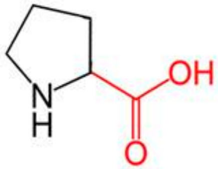 Pro	The WT residue is predicted (by the PHDsec DAS-server) to be located in an α-helix. Pro disrupts an α-helix when not located at one of the first 3 positions of that helix. In case of the mutation at hand, the helix will be disturbed and this can have severe effects on the structure of the protein. 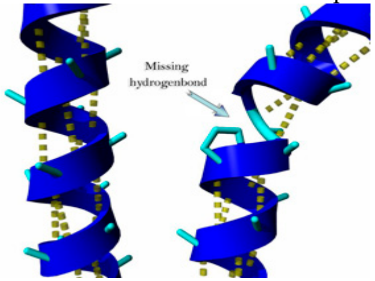 According to the PISA-database, the mutated residue is involved in a multimer contact. The PISA-database contains protein assemblies that are highly likely to be biologically relevant. The mutation introduces a smaller residue at this position. The new residue might be too small to make multimer contacts. A more hydrophobic residue is introduced here. Any hydrogen bond that could be made by the WT residue to other monomers will be lost now and affect the multimeric contacts.
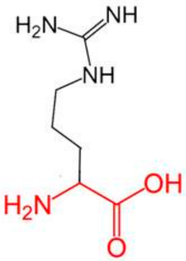 **Arg**	255	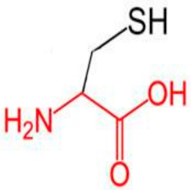 Cys	The WT residue forms a salt bridge with: Asp at position 262. The difference in charge will disturb the ionic interaction made by the original, WT residue. The charge of the WT residue is lost by this mutation. This can cause loss of interactions with other molecules.
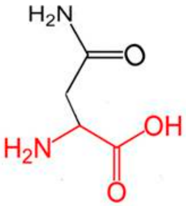 **Asn**	268	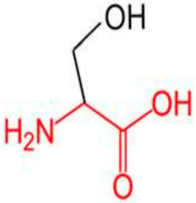 Ser	The mutation introduces a more hydrophobic residue at this position. This can result in loss of hydrogen bonds and/or disturb correct folding.

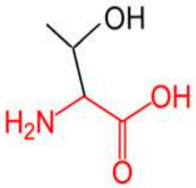 **Thr**	284	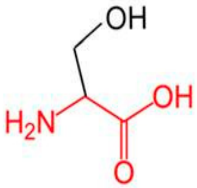 Ser	The residue is buried in the core of a domain. The differences between the WT and M residue might disturb the core structure of this domain. The mutation will cause an empty space in the core of the protein.
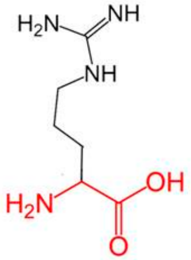 **Arg**	292	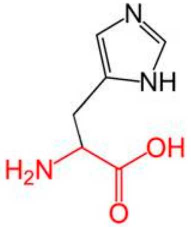 His	The WT residue forms a hydrogen bond with: Glu at position 422, Val at position 298. The size difference between WT and M residue makes that the new residue is not in the correct position to make the same hydrogen bond as the original WT residue did. The WT residue forms a salt bridge with: Glu at position 422. The difference in charge will disturb the ionic interaction made by the original, WT residue.
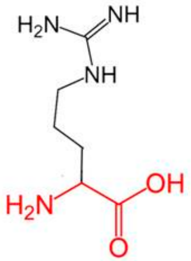 **Arg**	292	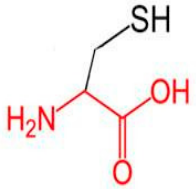 Cys	The WT residue forms a hydrogen bond with: Glu at position 422, Val at position 298. The size difference between WT and M residue makes that the new residue is not in the correct position to make the same hydrogen bond as the original WT residue did. The difference in hydrophobicity will affect hydrogen bond formation. The WT residue forms a salt bridge with: Glu at position 422. The difference in charge will disturb the ionic interaction made by the original, WT residue.
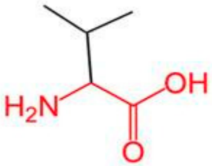 **Val**	298	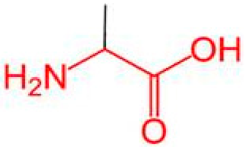 Ala	The residue is buried in the core of a domain. The differences between the WT and M residue might disturb the core structure of this domain. The mutation will cause an empty space in the core of the protein.

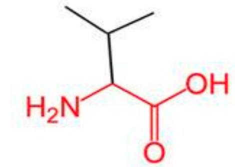 **Val**	298	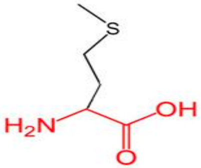 Met	The M residue is bigger than the WT residue. The WT residue was buried in the core of the protein. The M residue is bigger and probably will not fit.
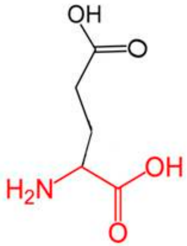 **Glu**	302	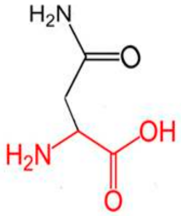 Lys	The WT residue forms a hydrogen bond with: Arg at position 289, Arg at position 289. The size difference between WT and M residue makes that the new residue is not in the correct position to make the same hydrogen bond as the original WT residue did. The WT residue forms a salt bridge with: Arg at position 289. The difference in charge will disturb the ionic interaction made by the original, WT residue. The residue is located on the surface of the protein, mutation of this residue can disturb interactions with other molecules or other parts of the protein.
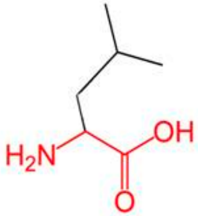 **Leu**	314	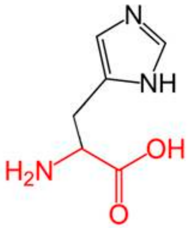 His	The mutation converts the WT residue in a residue that does not prefer α-helices as secondary structure. The residue is buried in the core of a domain. The differences between the WT and M residue might disturb the core structure of this domain.
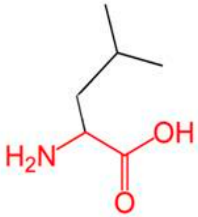 **Leu**	323	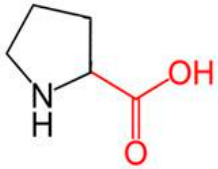 Pro	The M residue is smaller than the WT residue. This will cause a possible loss of external interactions.

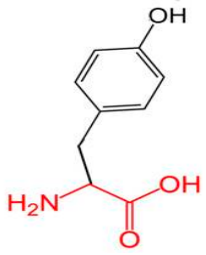 **Tyr**	338	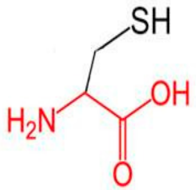 Cys	The M residue is smaller than the WT residue. The M residue is more hydrophobic than the WT residue. This will cause a possible loss of external interactions.
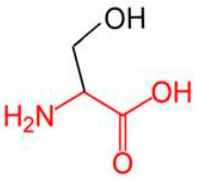 **Ser**	346	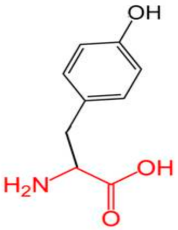 Tyr	The residue is located on the surface of the protein; mutation of this residue can disturb interactions with other molecules or other parts of the protein. The local conformation will be slightly destabilized.
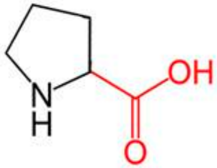 **Pro**	350	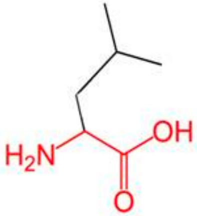 Leu	The residue is buried in the core of a domain. The differences between the WT and M residue might disturb the core structure of this domain. The WT residue is a Pro. Pros are known to be very rigid and therefore induce a special backbone conformation which might be required at this position. The mutation can disturb this special conformation.
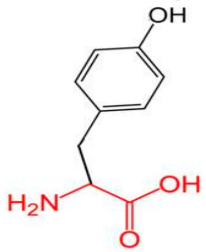 **Tyr**	352	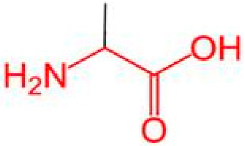 Ala	The M residue is smaller and more hydrophobic than the WT residue. The WT residue is predicted (by the PHDsec DAS-server) to be located in its preferred secondary structure, a β-strand. The M residue prefers to be in another secondary structure; therefore the local conformation will be slightly destabilized.

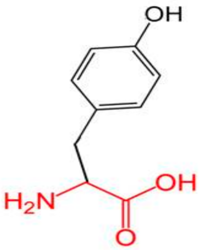 **Tyr**	352	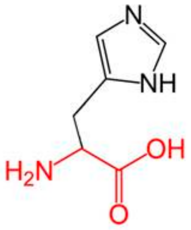 His	The M residue is smaller and more hydrophobic than the WT residue. The WT residue is predicted (by the PHDsec DAS-server) to be located in its preferred secondary structure, a β-strand. The M residue prefers to be in another secondary structure; therefore the local conformation will be slightly destabilized.
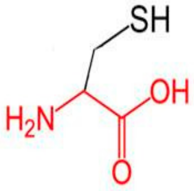 **Cys**	353	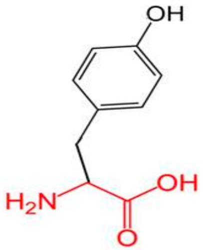 Tyr	The WT residue was buried in the core of the protein. The M residue is bigger and probably will not fit. The hydrophobicity of the WT and M residue differs. The mutation will cause loss of hydrophobic interactions in the core of the protein.
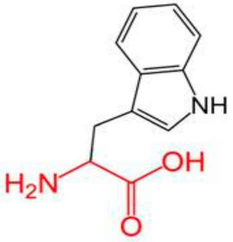 **Trp**	361	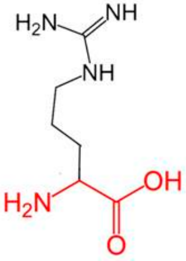 Arg	The WT residue forms a hydrogen bond with Gly at position 355. The size difference between WT and M residue makes that the new residue is not in the correct position to make the same hydrogen bond as the original WT residue did. The difference in hydrophobicity will affect hydrogen bond formation. The mutation introduces a charge at this position, this can cause repulsion between the M residue and neighboring residues.
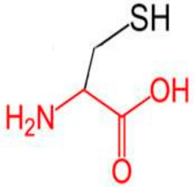 **Cys**	375	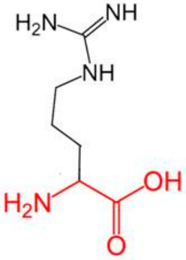 Arg	The M residue introduces a charge in a buried residue which can lead to protein folding problems. The WT residue was buried in the core of the protein. The M residue is bigger and probably will not fit.

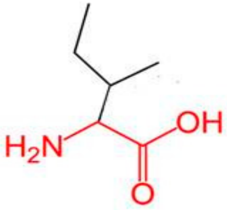 **Ile**	403	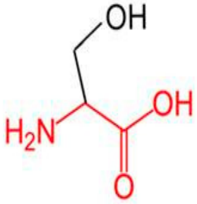 Ser	The residue is buried in the core of a domain. The differences between the WT and M residue might disturb the core structure of this domain. The M residue is smaller and less hydrophobic than the WT residue.
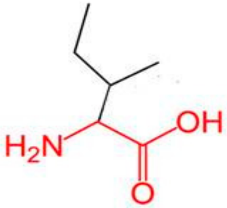 **Ile**	403	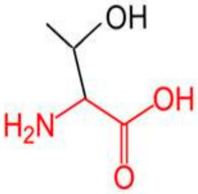 Thr	The M residue is smaller and less hydrophobic than the WT residue. The residue is buried in the core of a domain. The differences between the WT and M residue might disturb the core structure of this domain. The mutation will cause an empty space in the core of the protein.
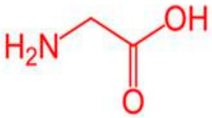 **Gly**	408	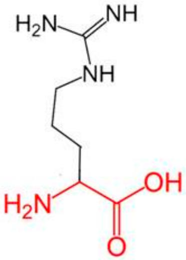 Arg	The WT residue is a Gly, the most flexible of all residues. This flexibility might be necessary for the protein's function. Mutation of this Gly can abolish this function. The torsion angles for this residue are unusual. Only Gly is flexible enough to make these torsion angles, mutation into another residue will force the local backbone into an incorrect conformation and will disturb the local structure.
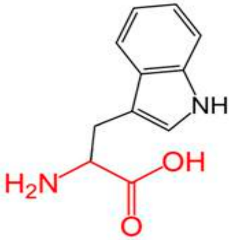 **Trp**	425	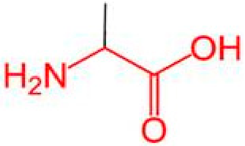 Ala	The WT residue forms a hydrogen bond with: Ile at position 415. The size difference between WT and M residue makes that the new residue is not in the correct position to make the same hydrogen bond as the original WT residue did. The mutation will cause an empty space in the core of the protein.

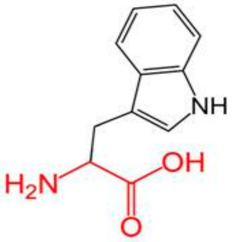 **Trp**	425	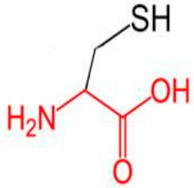 Cys	The WT residue forms a hydrogen bond with: Ile at position 415. The size difference between WT and M residue makes that the new residue is not in the correct position to make the same hydrogen bond as the original WT residue did. The mutation will cause an empty space in the core of the protein.
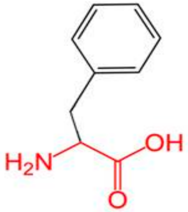 **Phe**	431	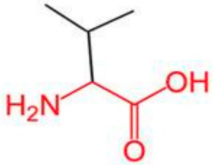 Val	The M residue is smaller than the WT residue. The mutation will cause an empty space in the core of the protein. The residue is buried in the core of a domain. The differences between the WT and M residue might disturb the core structure of this domain.

Table S4. Evaluating the effect of the amino acid mutations in SCD1 by HOPE sever. The distinct pattern and possible consequences of native and Mutant amino acid on protein structure based on size, charge, and hydrophobicity have been shown.

WT Amino Acid	Position	M Amino Acid	Characteristics and Possible Consequences
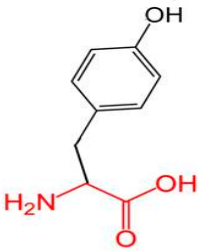 **Tyr**	41	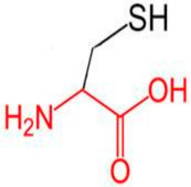 Cys	The M residue is smaller and more hydrophobic than the WT residue. This will cause a possible loss of external interactions. The mutation introduces a more hydrophobic residue at this position. This can result in loss of hydrogen bonds and/or disturb correct folding.

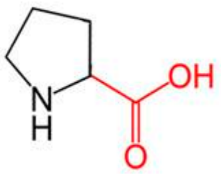 **Pro**	48	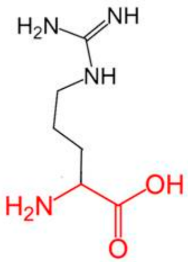 Arg	The M residue is bigger and less hydrophobic than the WT residue. The WT residue was neutral; the M residue is +vely charged. The WT residue was buried in the core of the protein. The M residue is bigger and probably will not fit. The WT residue is a Pro. Pros are known to be very rigid and therefore induce a special backbone conformation which might be required at this position. The mutation can disturb this special conformation. Hydrophobic interactions, either in the core of the protein or on the surface, will be lost.
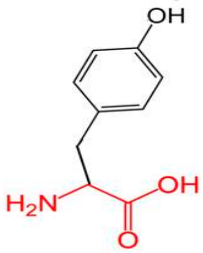 **Tyr**	55	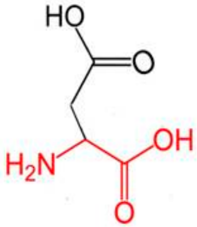 Asp	The M residue is smaller and less hydrophobic than the WT residue. The WT residue was neutral, the M residue is -vely charged. The mutation introduces a charge at this position; this can cause repulsion between the M residue and neighboring residues.
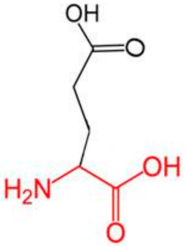 **Glu**	63	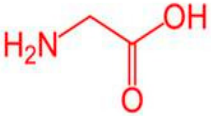 Gly	The M residue is smaller and more hydrophobic than the WT residue. The WT residue was -vely charged, the M residue is neutral. The mutated residue is not in direct contact with a ligand; however, the mutation could affect the local stability which in turn could affect the ligand-contacts made by one of the neighboring residues. The mutation introduces a Gly at this position. Glys are very flexible and can disturb the required rigidity of the protein at this position.
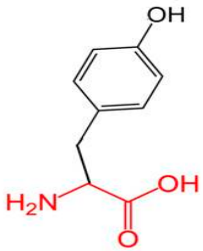 **Tyr**	88	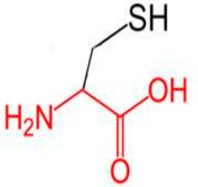 Cys	The M residue is smaller and more hydrophobic than the WT residue. This will cause a possible loss of external interactions. The mutated residue is located in a domain that is important for the activity of the protein and in contact with another domain that is also important for the activity. The interaction between these domains could be disturbed by the mutation, which might affect the function of the protein.

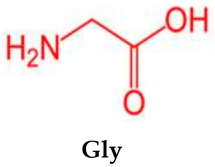 **Gly**	89	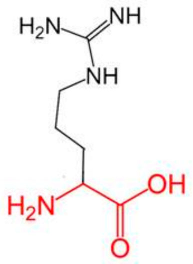 Arg	The M residue is bigger and less hydrophobic than the WT residue. The WT residue was neutral; the M residue is +vely charged. The WT residue is a Gly, the most flexible of all residues. This flexibility might be necessary for the protein's function. Mutation of this Gly can abolish this function. The torsion angles for this residue are unusual. Only Gly is flexible enough to make these torsion angles, mutation into another residue will force the local backbone into an incorrect conformation and will disturb the local structure.
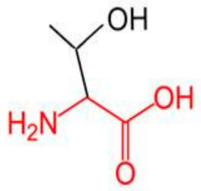 **Thr**	100	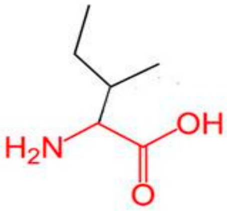 Ile	The M residue is bigger and more hydrophobic than the WT residue. The mutation will cause loss of hydrogen bonds in the core of the protein and as a result disturb correct folding. The WT residue was buried in the core of the protein. The M residue is bigger and probably will not fit.
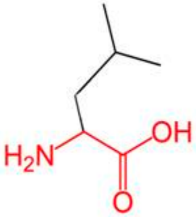 **Leu**	102	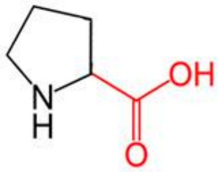 Pro	The M residue is smaller than the WT residue. The mutated residue is located in a domain that is important for the activity of the protein and in contact with another domain that is also important for the activity. The interaction between these domains could be disturbed by the mutation, which might affect the function of the protein.
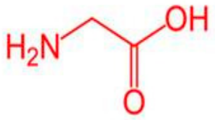 **Gly**	104	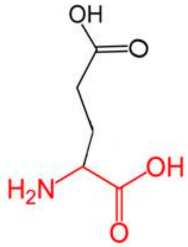 Glu	The M residue is bigger and less hydrophobic than the WT residue. The WT residue was neutral; the M residue is -vely charged. The WT residue is a Gly, the most flexible of all residues. This flexibility might be necessary for the protein's function. Mutation of this Gly can abolish this function. The WT residue was buried in the core of the protein. The M residue is bigger and probably will not fit.

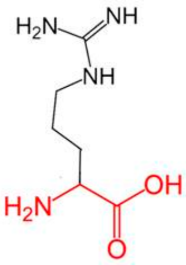 **Arg**	121	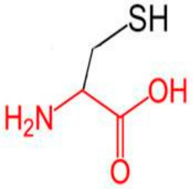 Cys	The M residue is smaller and more hydrophobic than the WT residue. The WT residue was +vely charged, the M residue is neutral. The mutated residue is not in contact with a metal; however, one of the neighboring residues does make a metal-contact that might be affected by the mutation in its vicinity. The mutated residue is not in direct contact with a ligand; however, the mutation could affect the local stability which in turn could affect the ligand-contacts made by one of the neighboring residues. The WT residue forms a hydrogen bond with: Glu at position 295, Thr at position 321, and Asn at position 318. The WT residue forms a salt bridge with: Glu at position 295, Asp at position 307 and Asp at position 325. The mutation will cause loss of hydrogen bonds in the core of the protein and as a result disturb correct folding.
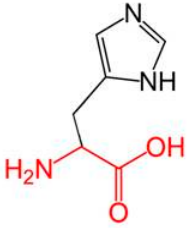 **His**	125	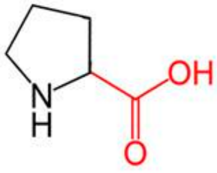 Pro	The M residue is smaller and more hydrophobic the WT residue. The size differences between the WT and M residue disturb the interaction with the metal-ion: "ZN". The WT residue forms a hydrogen bond with His at position 120, His at position 157, His at position 157 and His at position 301. The difference in hydrophobicity will affect hydrogen bond formation.
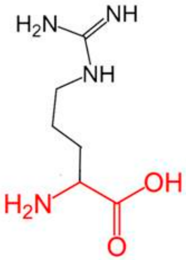 **Arg**	126	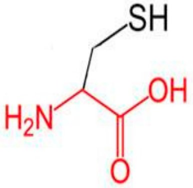 Cys	The M residue is smaller and more hydrophobic the WT residue. The WT residue was +vely charged; the M residue is neutral. The WT residue forms a hydrogen bond with Asp at position 204, Glu at position 208 and Asp at position 204. The difference in hydrophobicity will affect hydrogen bond formation. The WT residue forms a salt bridge with Asp at position 204 and Glu at position 208. The difference in charge will disturb the ionic interaction made by the original, WT residue.
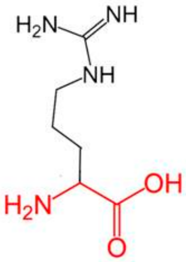 **Arg**	126	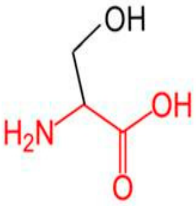 Ser	The M residue is smaller the WT residue. The WT residue was +vely charged; the M residue is neutral. The WT residue forms a hydrogen bond with Asp at position 204, Glu at position 208 and Asp at position 204. The difference in hydrophobicity will affect hydrogen bond formation. The WT residue forms a salt bridge with Asp at position 204 and Glu at position 208. The difference in charge will disturb the ionic interaction made by the original, WT residue.

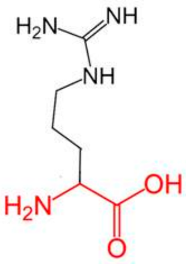 **Arg**	131	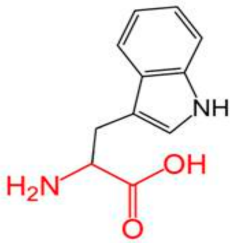 Trp	The M residue is bigger and more hydrophobic than the WT residue. The WT residue was +vely; the M residue is neutral. The residue is located on the surface of the protein; mutation of this residue can disturb interactions with other molecules or other parts of the protein.
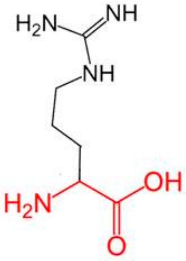 **Arg**	135	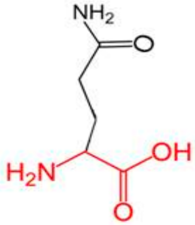 Gln	The M residue is smaller than the WT residue. The WT residue was +vely charged; the M residue is neutral. The WT residue forms a salt bridge with Glu at position 208. The difference in charge will disturb the ionic interaction made by the original, WT residue.
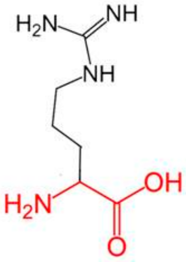 **Arg**	135	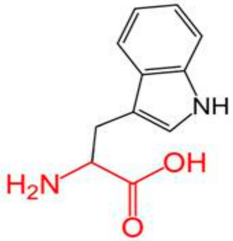 Trp	The M residue is bigger and more hydrophobic than the WT residue. The WT residue was +vely; the M residue is neutral. The residue is located on the surface of the protein; mutation of this residue can disturb interactions with other molecules or other parts of the protein.
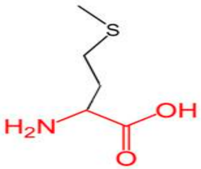 **Met**	144	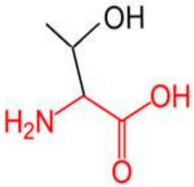 Thr	The M residue is smaller and less hydrophobic than the WT residue. The mutation might cause loss of hydrophobic interactions with other molecules on the surface of the protein.

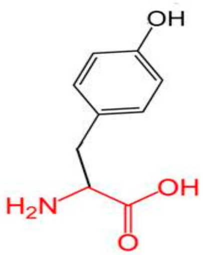 **Tyr**	151	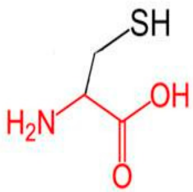 Cys	The M residue is smaller and more hydrophobic than the WT residue. The WT residue forms a hydrogen bond with Glu at position 206. The mutation will cause loss of hydrogen bonds in the core of the protein and as a result disturb correct folding.
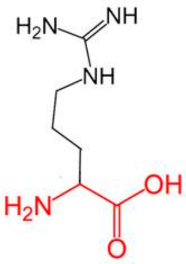 **Arg**	188	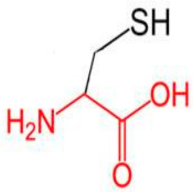 Cys	The M residue is smaller and more hydrophobic the WT residue. The WT residue was +vely charged; the M residue is neutral. The WT residue forms a salt bridge with Glu at position 70. The difference in charge will disturb the ionic interaction made by the original, WT residue. The difference in hydrophobicity will affect hydrogen bond formation.
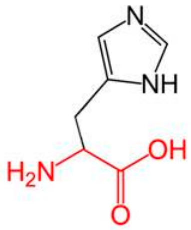 **His**	190	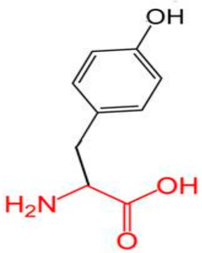 Tyr	The M residue is bigger and more hydrophobic the WT residue. The WT residue forms a hydrogen bond with Ala at position 192. The residue is buried in the core of a domain. The differences between the WT and M residue might disturb the core structure of this domain. The mutation will cause loss of hydrogen bonds in the core of the protein and as a result disturb correct folding.
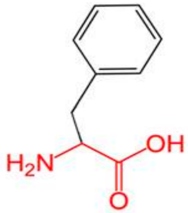 **Phe**	213	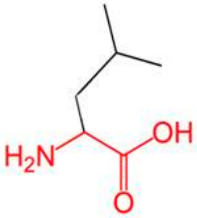 Leu	The M residue is smaller than the WT residue. This will cause a possible loss of external interactions.

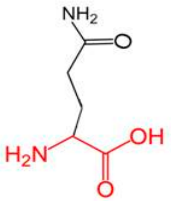 **Gln**	214	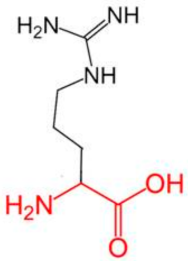 Arg	The M residue is bigger than the WT residue. The WT residue was neutral; the M residue is +vely. The mutated residue is not in direct contact with a ligand; however, the mutation could affect the local stability which in turn could affect the ligand-contacts made by one of the neighboring residues. The WT residue forms a hydrogen bond with Val at position 150. The size difference between WT and M residue makes that the new residue is not in the correct position to make the same hydrogen bond as the original WT residue did. The WT residue was buried in the core of the protein. The M residue is bigger and probably will not fit.
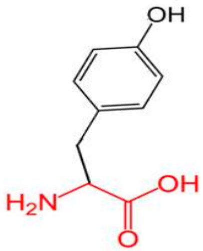 **Tyr**	218	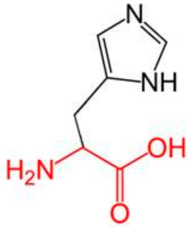 His	The M residue is smaller and less hydrophobic than the WT residue. The WT residue forms a hydrogen bond with Gln at position 147. The difference in hydrophobicity will affect hydrogen bond formation.
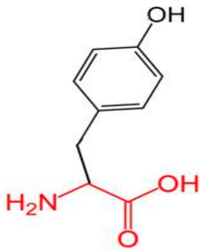 **Tyr**	218	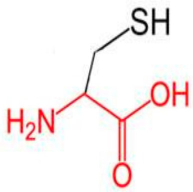 Cys	The M residue is smaller and more hydrophobic than the WT residue. The WT residue forms a hydrogen bond with Gln at position 147. The difference in hydrophobicity will affect hydrogen bond formation.
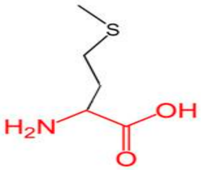 **Met**	225	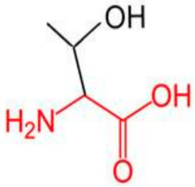 Thr	The M residue is smaller and less hydrophobic than the WT residue. The mutation might cause loss of hydrophobic interactions with other molecules on the surface of the protein.

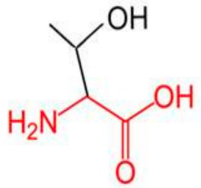 **Thr**	231	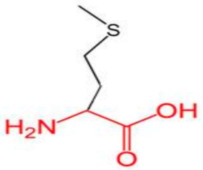 Met	The M residue is bigger and more hydrophobic than the WT residue. The WT residue forms a hydrogen bond with Phe at position 227 and Thr at position 250. The mutation will cause loss of hydrogen bonds in the core of the protein and as a result disturb correct folding.
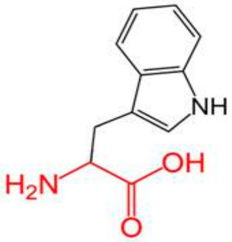 **Trp**	238	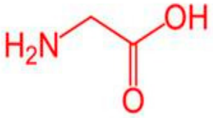 Gly	The M residue is smaller and more hydrophobic than the WT residue. The mutation introduces a Gly at this position. Glys are very flexible and can disturb the required rigidity of the protein at this position.
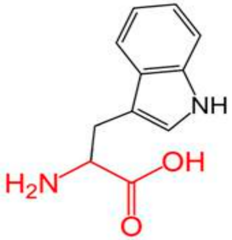 **Trp**	238	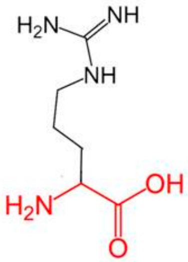 Arg	The M residue is smaller and more hydrophobic than the WT residue. The WT residue was neutral, the M residue is +vely charged. The mutation is located within a topological domain. The mutation might disrupt this topological domain.
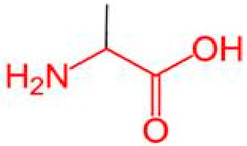 **Ala**	249	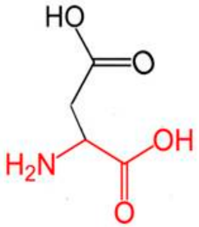 Asp	The M residue is bigger and less hydrophobic than the WT residue. The WT residue was neutral, the M residue is -vely charged. The residue is buried in the core of a domain. The differences between the WT and M residue might disturb the core structure of this domain.

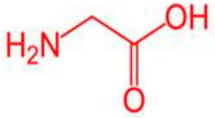 **Gly**	272	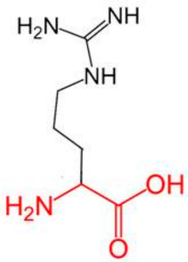 Arg	The M residue is bigger and less hydrophobic than the WT residue. The WT residue was neutral, the M residue is +vely charged. The WT residue is a Gly, the most flexible of all residues. This flexibility might be necessary for the protein's function. Mutation of this Gly can abolish this function. The residue is located on the surface of the protein; mutation of this residue can disturb interactions with other molecules or other parts of the protein. The torsion angles for this residue are unusual. Only Gly is flexible enough to make these torsion angles, mutation into another residue will force the local backbone into an incorrect conformation and will disturb the local structure.
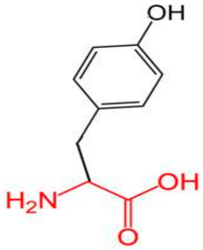 **Tyr**	273	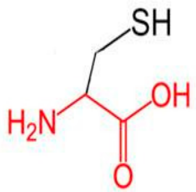 Cys	The M residue is smaller and more hydrophobic than the WT residue. The WT residue is annotated to be located in its preferred secondary structure, a β-strand. The M residue prefers to be in another secondary structure; therefore the local conformation will be slightly destabilized.
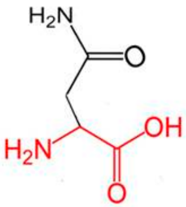 **Asn**	279	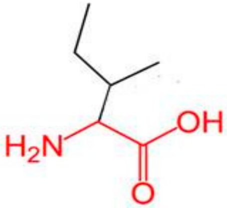 Ile	The M residue is smaller and more hydrophobic than the WT residue.
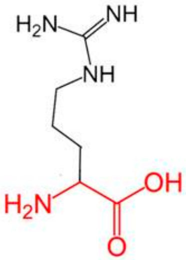 **Arg**	283	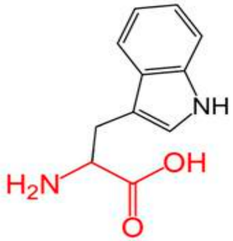 Trp	The M residue is bigger and more hydrophobic than the WT residue. The WT residue was +vely charged, the M residue is neutral. The mutated residue is not in contact with a metal, however, one of the neighboring residues does make a metal-contact that might be affected by the mutation in its vicinity. The WT residue forms a salt bridge with Glu at position 165. The difference in charge will disturb the ionic interaction made by the original, WT residue. The residue is located on the surface of the protein; mutation of this residue can disturb interactions with other molecules or other parts of the protein.

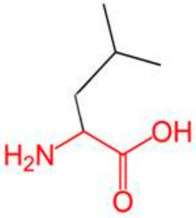 **Leu**	290	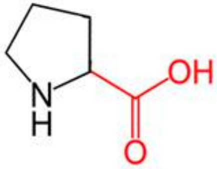 Pro	The M residue is smaller than the WT residue. The WT residue is located in a region annotated to form an α-helix. Pro disrupts an α-helix when not located at one of the first 3 positions of that helix. In case of the mutation at hand, the helix will be disturbed and this can have severe effects on the structure of the protein. 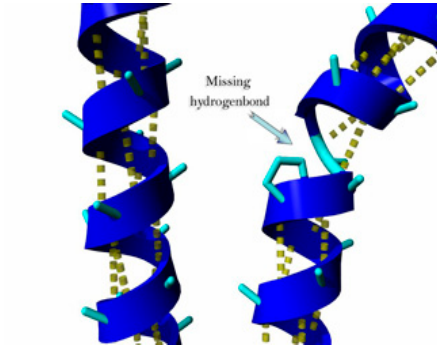
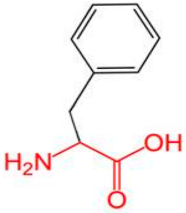 **Phe**	323	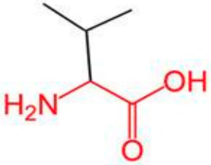 Val	The M residue is smaller than the WT residue. This will cause a possible loss of external interactions.
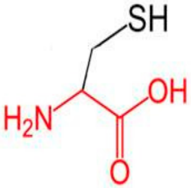 **Cys**	326	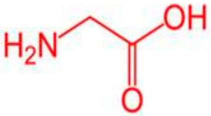 Gly	The M residue is smaller and less hydrophobic than the WT residue. The mutation introduces a Gly at this position. Glys are very flexible and can disturb the required rigidity of the protein at this position.
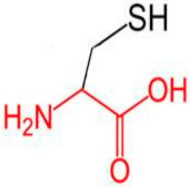 **Cys**	326	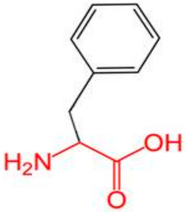 Phe	The M residue is bigger than the WT residue. The residue is located on the surface of the protein; mutation of this residue can disturb interactions with other molecules or other parts of the protein.

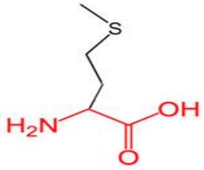 **Met**	327	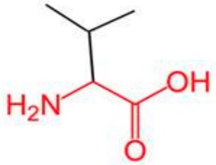 Val	The M residue is smaller than the WT residue. The WT residue is located in a region annotated to form an α-helix. The mutation converts the WT residue in a residue that does not prefer α-helices as secondary structure.
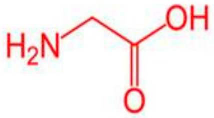 **Gly**	331	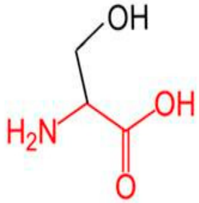 Ser	The M residue is bigger than the WT residue. The WT residue is a Gly, the most flexible of all residues. This flexibility might be necessary for the protein's function. Mutation of this Gly can abolish this function. The residue is located on the surface of the protein; mutation of this residue can disturb interactions with other molecules or other parts of the protein. The torsion angles for this residue are unusual. Only Gly is flexible enough to make these torsion angles, mutation into another residue will force the local backbone into an incorrect conformation and will disturb the local structure.
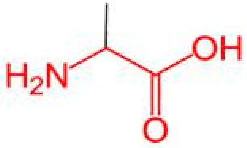 **Ala**	333	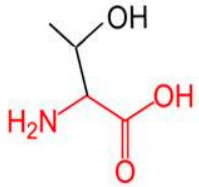 Thr	The M residue is bigger and more hydrophobic than the WT residue. The residue is buried in the core of a domain. The differences between the WT and M residue might disturb the core structure of this domain. The WT residue was buried in the core of the protein. The M residue is bigger and probably will not fit.
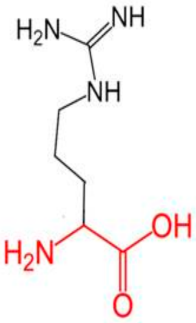 **Arg**	336	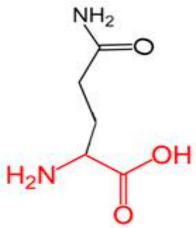 Gln	The M residue is smaller than the WT residue. The WT residue was +vely charged, the M residue is neutral. The WT residue forms a hydrogen bond with Asp at position 325. The size difference between WT and M residue makes that the new residue is not in the correct position to make the same hydrogen bond as the original WT residue did. The WT residue forms a salt bridge with Asp at position 325 and Asp at position 335. The charge of the WT residue is lost by this mutation. This can cause loss of interactions with other molecules.

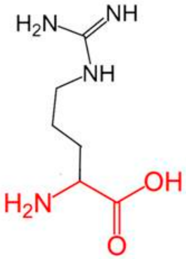 **Arg**	336	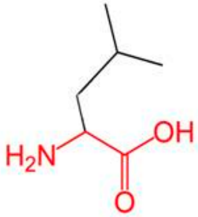 Leu	The M residue is smaller and more hydrophobic than the WT residue. The WT residue was +vely charged, the M residue is neutral. The WT residue forms a hydrogen bond with Asp at position 325. The size difference between WT and M residue makes that the new residue is not in the correct position to make the same hydrogen bond as the original WT residue did. The difference in hydrophobicity will affect hydrogen bond formation. The WT residue forms a salt bridge with Asp at position 325 and Asp at position 335. The difference in charge will disturb the ionic interaction made by the original, WT residue.
